# Automated Extraction of Postoperative Cancer Recurrence and Metastasis From Computed Tomography (CT) Reports: Semisupervised Deep Learning Study

**DOI:** 10.2196/92937

**Published:** 2026-09-08

**Authors:** Wonkeun Jo, Bumwoo Park, Ji-Hoon Sim

**Affiliations:** 1 Biomedical Engineering Research Center, Asan Institute for Life Sciences, Asan Medical Center, Seoul, Republic of Korea; 2 Center for Space Exploration Technology, Division of Space Exploration, Korea Astronomy and Space Science Institute, Daejeon, Republic of Korea; 3 Department of Information Medicine, Asan Medical Center, Seoul, Republic of Korea; 4 Department of Anesthesiology and Pain Medicine, University of Ulsan College of Medicine, Asan Medical Center, 88, Olympic-ro 43-Gil, Songpa-gu, Seoul, 05505, Republic of Korea, 1 1688-7575

**Keywords:** computed tomography, radiology information systems, neoplasm recurrence, neoplasm metastasis, natural language processing, uncertainty, retrospective studies, deep learning

## Abstract

**Background:**

Perioperative computed tomography (CT) imaging is essential for detecting postoperative recurrence and metastasis in cancer patients. However, large-scale automated extraction of oncological outcomes from CT reports remains limited by the unstructured nature of report text and wide variability in reporting styles. Radiology reports frequently contain linguistic ambiguities, including negations, hedging, and expressions conveying diagnostic uncertainty (eg, “cannot exclude recurrence” or “possibly metastatic”). Manual review is labor-intensive and constrains consistent extraction at scale. The inability to systematically account for diagnostic uncertainty represents a major barrier to reliable automated surveillance systems.

**Objective:**

This study aimed to develop a semisupervised deep learning (DL) classification framework that explicitly captures diagnostic uncertainty by classifying postoperative recurrence and metastasis into 3 categories (positive, negative, and uncertain).

**Methods:**

This retrospective study analyzed 288,076 postoperative CT reports from 86,083 cancer surgery patients at Asan Medical Center (2014‐2021). After exact-match deduplication, model training and evaluation used 17,846 unique reports for recurrence and 63,766 for metastasis. Preprocessing identified presumed negatives through keyword filtering and unsupervised clustering. The semisupervised framework incorporated human-in-the-loop validation across 3 cycles—with clinicians reviewing approximately 2000 samples per cycle (<1% of total reports)—and integrated rule-based algorithms (RAs) and medical BERT (MedEmbed and PubMedBERT). A report-level train-validation split was used, as preprocessing reduces each report to sentence-level fragments that preclude patient-level linkage. Performance was evaluated against RAs and multiple BERT variants under both naive and simulated real-world class distributions. Maximum mean discrepancy testing confirmed distributional integrity of the sampled data. Model interpretability was assessed using Integrated Gradients.

**Results:**

The cohort included 11 cancer types, predominantly gastrointestinal (28,516/86,083, 33.1%), hepatobiliary and pancreas (14,715/86,083, 17.1%), and genitourinary (12,959/86,083, 15.1%). Under simulated conditions, PubMedBERT achieved 92.58% accuracy for recurrence in the multiclass, and MedEmbed achieved 93.25% for metastasis in the binary class. The framework achieved accuracies of 97.33% (multiclass) and 99.33% (binary class) for recurrence and 95.00% (multiclass) and 96.67% (binary class) for metastasis, compared with human intrarater consistencies of 96.88% and 93.80% (recurrence and metastasis, respectively, for multiclass), reflecting concordance with the clinician-derived consensus standard. The framework captured diagnostic uncertainty in 1.4% of recurrence cases and 6.9% of metastasis cases. Notably, the RA outperformed several sophisticated DL models in metastasis classification.

**Conclusions:**

The proposed framework achieves clinician-concordant classification across the full 288,076-report corpus while requiring minimal expert annotation (<1% of reports). By explicitly modeling diagnostic uncertainty and combining rule-based and DL approaches, it demonstrates the potential for automated cancer surveillance and clinical decision support in real-world settings; however, generalizability to other institutions requires prospective multicenter validation.

## Introduction

Perioperative computed tomography (CT) imaging plays a crucial role in diagnosing and monitoring patients undergoing cancer surgery, and radiology reports provide essential information for detecting recurrence or distant metastasis [1]. Although individual CT examinations are interpreted with high accuracy in clinical practice, the large-scale use of radiology reports for research, quality monitoring, or automated surveillance remains limited. This limitation primarily stems from the unstructured nature of report text and wide variability in reporting styles [2-4]. These challenges, which include substantial institutional and stylistic variation, undermine the reliability of automated information extraction across diverse clinical environments [5]. Consequently, manual review remains highly labor-intensive and constrains the consistent, large-scale extraction of oncologic outcomes [4,6].

Radiology reports frequently contain linguistic ambiguities such as negations, hedging, and expressions that convey diagnostic uncertainty (for example, “cannot exclude recurrence” or “possibly metastatic”) [7-9]. In clinical practice, such language is typically addressed through conservative interpretation. Recent studies on extracting oncologic outcomes from free-text radiology reports have demonstrated that nonstandardized and ambiguous expressions substantially complicate automated detection [10,11]. As interest in population-level postoperative surveillance and automated extraction of oncologic outcomes continues to grow, the inability to systematically account for diagnostic uncertainty remains a major barrier to the reliability of large-scale monitoring systems [12].

To address these gaps, we proposed a language model (LM)–based system that classifies postoperative CT impressions by assigning both recurrence and metastasis into 1 of 3 categories (positive, negative, and uncertain). This design explicitly incorporates the ambiguous expressions that commonly appear in real-world radiology reporting [9,11]. By leveraging advances in biomedical language representation and pretrained deep learning (DL) models [13-15], together with uncertainty-aware approaches reported in natural language processing (NLP), the proposed framework aimed to improve classification consistency and accuracy in heterogeneous postoperative CT reports [16].

## Methods

### Study Design

This retrospective study analyzed 288,076 postoperative CT radiology reports from 86,083 patients who underwent cancer surgery at Asan Medical Center, Seoul, Republic of Korea. To address the lack of manual annotations for recurrence or metastasis, we developed a semisupervised learning framework incorporating a human-in-the-loop (HL) approach to enable scalable labeling. This approach is considered semisupervised because clinician-reviewed gold standard labels were available only for positive and uncertain class samples, while the remaining negative samples were incorporated as weakly labeled samples during training. In this framework, clinicians initially provided raw radiology reports and reviewed the model’s predictions, providing targeted feedback on ambiguous or clinically relevant cases. Guided by this feedback, the model was iteratively refined to improve classification performance. The finalized framework yielded 2 primary outputs: (1) a trained model capable of predicting recurrence and metastasis for newly acquired postoperative CT reports and (2) predicted labels for the entire historical cohort of reports.

### Framework

**Figure 1.**
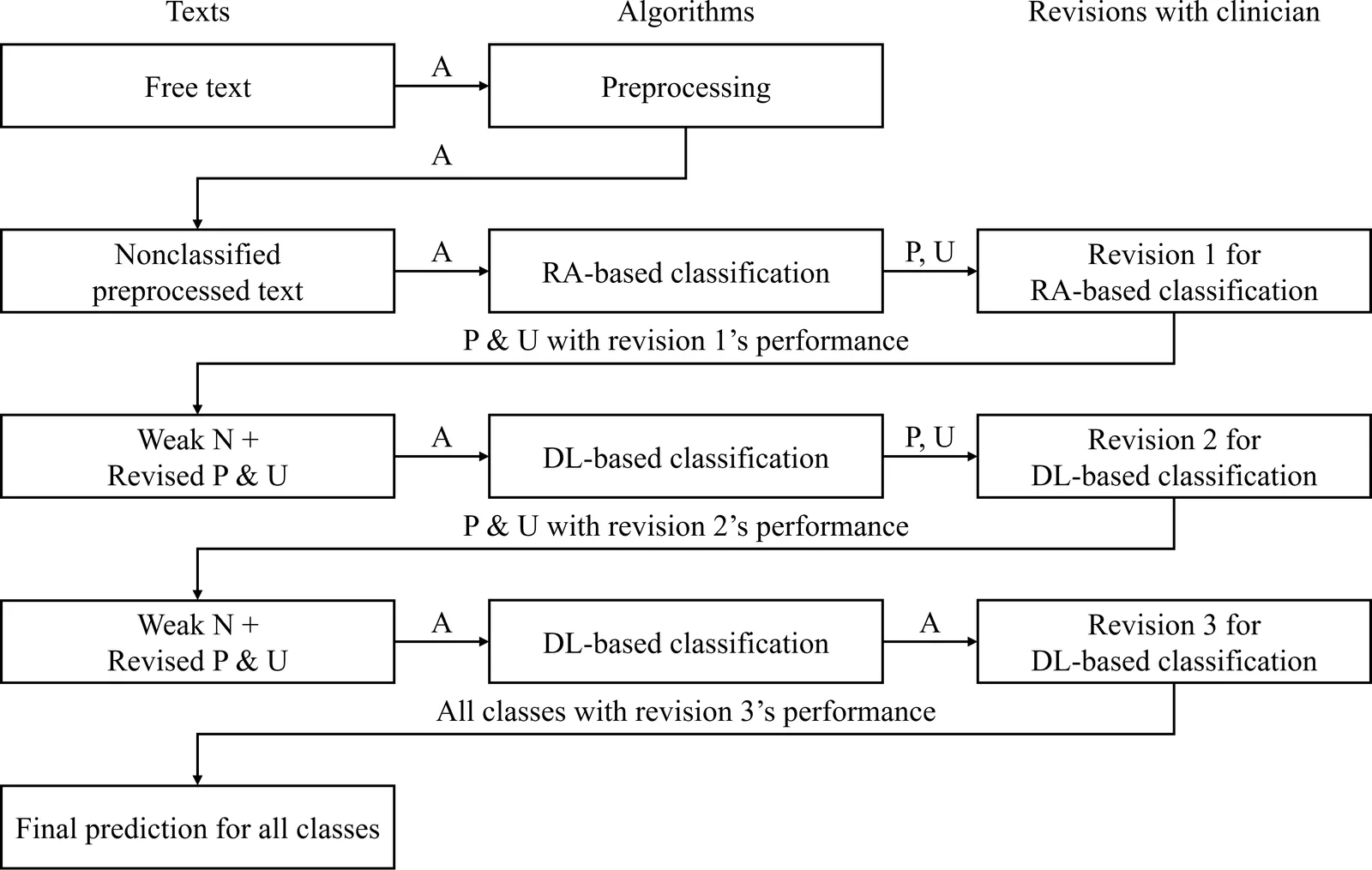
Human-in-the-loop schedule for iterative label generation from 288,076 postoperative computed tomography (CT) radiology reports collected at Asan Medical Center (2014‐2021) across 3 revision cycles, with only positive and uncertain conditions reviewed by clinicians in revisions 1 and 2 and all classes including negative cases reviewed in revision 3 to establish the final performance benchmark. A: algorithm-generated predictions applied to all classes; DL: deep learning; N: weak negatives identified through hybrid clustering; P: positive cases forwarded for clinician review; RA: rule-based algorithm; U: uncertain cases forwarded for clinician review.

The proposed framework was built around an HL schedule, in which clinician review and model training were iteratively coupled to generate reliable labels from large-scale unlabeled CT radiology reports (Figure 1). To support this iterative process, the framework incorporated 3 preparatory components: preprocessing, presumed negative labeling, and classification.

### Preprocessing

Raw CT radiology reports underwent 2 sequential steps. First, report text was cleaned by removing formatting artifacts such as excessive line spacing and hyphen-based line separators that commonly appear in clinical documentation. Second, the cleaned reports were filtered using keyword matching (“recur” and “metas”) to identify reports relevant to postoperative recurrence or metastasis. These keywords were applied as word stems rather than exact matches; thus, “recur” captured recurrence and recurrent disease, while “metas” captured metastasis, metastatic lesion, and metastases. In clinical practice, recurrence and metastasis represent critical postoperative findings that radiologists are expected to document explicitly, as failure to use standard terminology for critical findings may affect the completeness of clinical documentation and downstream management decisions. This assumption is clinically reasonable given institutional documentation standards, where radiologists are expected to use explicit terminology for critical findings; however, cases described using synonymous or indirect language may be inadvertently excluded. Accordingly, reports without a keyword match were designated as “presumed negatives” and excluded from subsequent classification.

To quantify the potential loss of positive cases during preprocessing, we performed a manual audit of 300 randomly selected keyword-excluded reports from the presumed-negative pool. Notably, the preprocessing steps substantially simplified report text, reducing patient-specific linguistic variation across reports. As a result, consistent patterns were observed across patients rather than within individual patient trajectories, justifying the use of report-level rather than patient-level data splitting in subsequent analyses. We also identified a substantial number of exact duplicate entries among the preprocessed samples. Accordingly, exact-match deduplication was performed across all samples separately for each prediction target (recurrence and metastasis) prior to train/validation splitting, thereby preventing data leakage introduced by report-level data splitting. The Preprocessing Analysis section describes the kind of preprocessed samples (prior to deduplication) that would be used in model learning, and the details of preprocessing and preprocessing efficiency are described in Multimedia Appendix 1.

### Weak Negative Labeling

In the absence of predefined labels in the initial dataset, we implemented a semisupervised clustering strategy consistent with prior work [17,18] to identify samples likely to represent “weak-negative” cases relative to other categories. The preprocessed reports were analyzed under 2 class-assumption approaches: a rule-based algorithm (RA) and a BERT-based decomposition and clustering method using UMAP-DBSCAN [19-21]. Reports were tagged as weak-negative when their corresponding clusters contained unique cases that were consistently prelabeled as negative prior to clinician revision. These weak-negative samples were excluded from all HL revision cycles to minimize clinician burden. Labeling criteria are detailed in the Details of Labeling section in Multimedia Appendix 1.

### Classification

Classification was performed using the RA and medical BERT models. The RA used regular expressions and clinical keywords (eg, “rule out,” Table S1 in Multimedia Appendix 1) to generate weak labels, establishing a baseline annotation for radiology reports. In RA, expressions such as “rule out” were consistently assigned an uncertain label regardless of whether they reflected a diagnostic finding or a clinical indication for the examination. However, the DL models were trained exclusively on clinician-reviewed gold standard labels, including uncertain cases verified through the HL revision cycles, thereby mitigating the impact of this initial labeling ambiguity on the final model. For classification, we deployed medical BERT, such as MedEmbed BERT [22] and PubMedBERT [23]. The Details of Text Classification section in Multimedia Appendix 1 explains the structure of the model and training loss.

### Dataset

#### Clinical Metadata

Patient demographic and clinical characteristics, including sex, age at surgery, and cancer type, were extracted from the institutional surgical registry maintained at Asan Medical Center. This registry was constructed and is continuously curated by the clinical team through systematic linkage with the hospital’s electronic medical records system. Because all patients in the cohort had undergone surgery at Asan Medical Center and subsequently received postoperative CT imaging with formal radiology reporting within the same institution, each radiology report could be unambiguously matched to a corresponding surgical record. Sex and age at surgery were complete for all 86,083 patients. Cancer type was defined according to the primary surgical diagnosis recorded in the surgical registry. For a small number of patients, the primary cancer type could not be assigned and was categorized as “missing.” These patients were retained in all analyses to preserve the full cohort. Age group was assigned based on the patient’s age at the time of inspection. Demographic subgroup analyses presented in the Results section were derived exclusively from these registry-linked variables.

#### Annotations

All annotations were performed by a single attending physician and faculty member specializing in anesthesiology and pain medicine with more than 15 years of clinical experience at Asan Medical Center. For ambiguous cases that were difficult to classify, consultation was sought from a faculty radiologist at Asan Medical Center. At each revision cycle, clinician review was prioritized for positive and uncertain cases to maximize annotation efficiency [24]. In revision 1, samples were obtained through stratified random sampling from 1000 temporary positive labels per target classified by the RA. In revision 2, stratified random sampling was performed using predictions from the DL model fine-tuned on revision 1 gold standard labels, with 500 positive and uncertain samples selected per target. In revision 3, all predicted classes including negative cases were reviewed to finalize the gold standard, with 100 samples per class per target, totaling 600 clinician-reviewed samples across both targets.

### Experiments

#### Statistical Validation of Dataset

To confirm that our gold standard dataset maintained distributional consistency with the broader clinical population, we used the maximum mean discrepancy (MMD) [25] test (see the Details of Statistical Testing section in Multimedia Appendix 1.) In revision 3, for each target and class, we resampled 100 unlabeled reports. Each separated group was evaluated using the MMD test with a permutation procedure against 100 randomly sampled reports from the full dataset. This process was repeated 500 times for all classes across the 2 targets.

#### Evaluation Strategy

**Figure 2.**
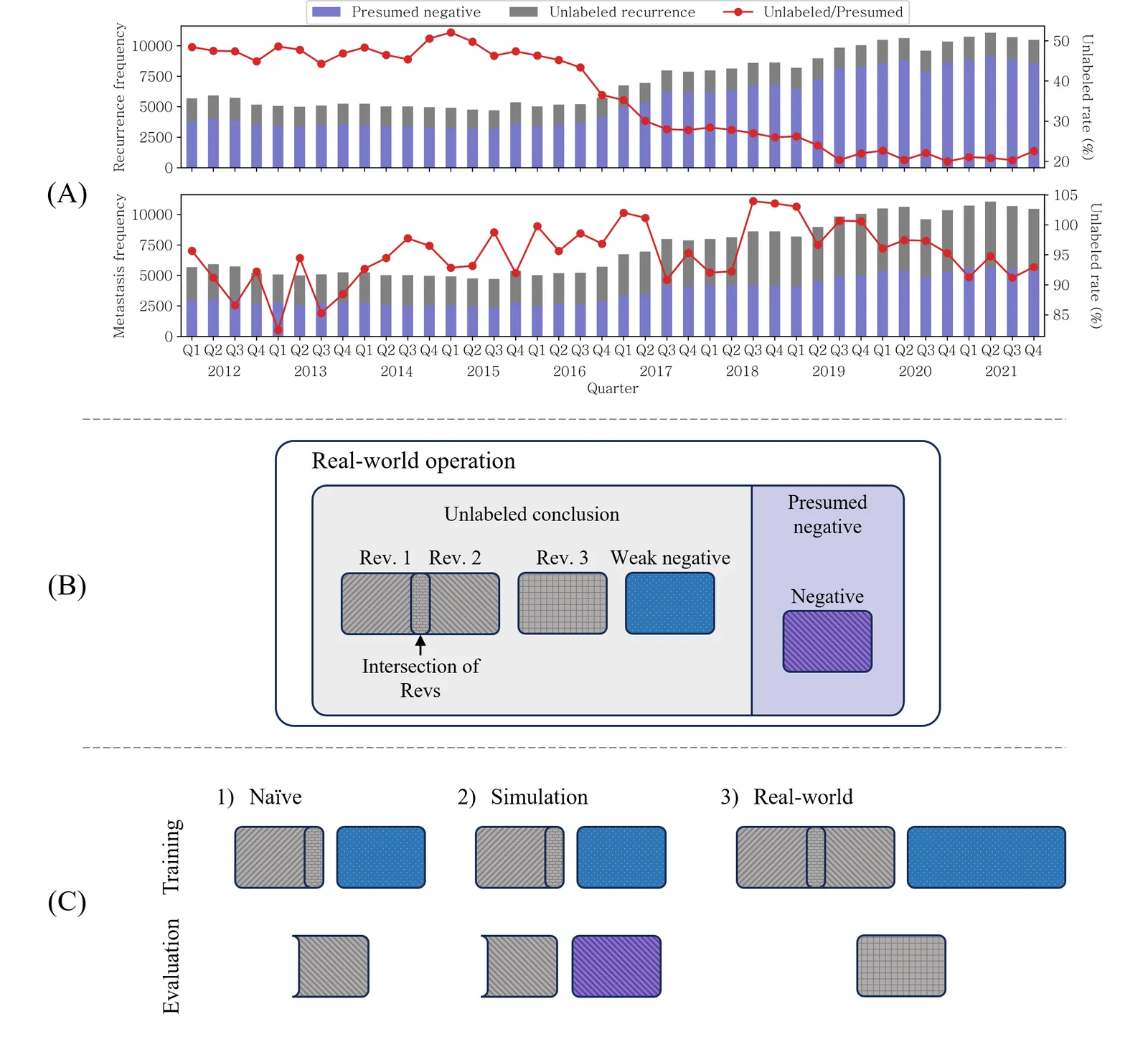
Dataset construction and evaluation strategy for postoperative computed tomography (CT) radiology reports, including the (A) quarterly distribution of presumed negative and unlabeled reports for recurrence and metastasis, with the unlabeled-to-presumed ratio shown as a red line; (B) a composition of the full dataset including clinician-reviewed samples from 3 revision (rev.) cycles, weak negative samples, and presumed negatives; and (C) the following 3 evaluation scenarios differing in the composition of training and evaluation sets: naive, simulation, and real world.

Although positive and uncertain cases were deliberately overrepresented in the clinician-reviewed samples, distributional consistency with the broader clinical population was assessed using statistical testing [25]. Therefore, model evaluation was performed under 3 complementary settings to account for this sampling imbalance. The evaluation strategy is illustrated in Figure 2.

The first was a naive evaluation that preserved the label distribution of the training data. The second was a simulation-based evaluation in which resampled presumed negative samples were incorporated to approximate real-world clinical distributions. The third was a real-world evaluation in which the model trained on revisions 1 and 2 generated predictions for all classes and agreement with clinician-reviewed labels in revision 3 was assessed. A small subset of reports received inconsistent labels across revisions 1 and 2, and the proportion of these discrepancies was used to estimate the human annotation error rate, which was regarded as an upper bound of achievable model performance.

For representative model selection, we adopted a strategy in which the model was trained using all gold standard labels accumulated up to that point and evaluated on the subsequent revision set. Models were trained on revision 1 alone and on the combined dataset from revisions 1 and 2. Cross-validation was performed using samples unique to revision 2, excluding overlapping cases. Finally, a model trained on the combined dataset from revisions 1 and 2 was evaluated on revision 3 to assess whether model performance approached the estimated human annotation accuracy.

#### Decision Interpretation

For clinical interpretability, the integrated gradients (IG) method was used to attribute predictions to specific features [26]. By integrating gradients along a path from a neutral baseline, the IG method avoids gradient saturation and satisfies the axiom of completeness. Positive IG scores highlight tokens favoring a class (eg, “metastasis”), whereas negative scores indicate suppressive terms (eg, “no evidence of”).

#### Experimental Setup

To ensure robust convergence, we used a stratified 9:1 train:validation split at the report level, as the preprocessing pipeline reduces each report to sentence-level representations that preclude patient-level linkage (see the Preprocessing section). We selected the model checkpoint that achieved the highest macro *F*_1_-score on the validation set. All experiments were conducted using a fixed random seed of 42 to ensure reproducibility. Predictive performance for LM selection was evaluated using 10-fold cross-validation with the symmetric difference of revisions 1 and 2. All LMs are listed in Table S3 in Multimedia Appendix 1. Given the class imbalance, accuracy, macro *F*_1_-score, and weighted *F*_1_-score were used as primary evaluation metrics to ensure balanced performance across all classes, including the minority uncertain class. For binary classification tasks, the *F*_1_-score for the positive class was additionally reported.

Performance was evaluated under 3 classification settings. The first was a multiclass setting (positive, negative, and uncertain). The second was a binary setting in which uncertain cases were grouped with negative cases (positive vs others; hereafter referred to as binary conservative), representing the clinically conservative assumption that unconfirmed findings are treated as nonevents. The third was a binary setting in which cases with uncertain labels—either in the gold standard or model prediction—were excluded entirely (positive vs negative; hereafter referred to as binary exclusive), enabling assessment of model performance on unambiguous cases only. Together, binary conservative and binary exclusive serve as sensitivity analyses to examine how the handling of diagnostic uncertainty affects classification performance, as uncertain findings grouped with negative findings may lead to missed follow-ups in oncologic surveillance contexts. Detailed descriptions of the experimental environment, hyperparameter settings, and metric definitions are provided in the Details of Experimental Setup section in Multimedia Appendix 1.

### Ethical Considerations

This retrospective cohort study was approved by the institutional review board of Asan Medical Center (2024‐1090), which waived the requirement for informed consent because the analysis relied exclusively on preexisting clinical and imaging data. To protect patient privacy, all datasets were deidentified; specifically, text data were preprocessed to retain only sentences containing keywords related to metastasis or recurrence, rendering individual identification from the resultant fragments impossible. No compensation was provided to any patients. Furthermore, we confirmed that all images in this manuscript were fully anonymized and contained no identifiable information.

## Results

### Dataset Analysis

**Figure 3.**
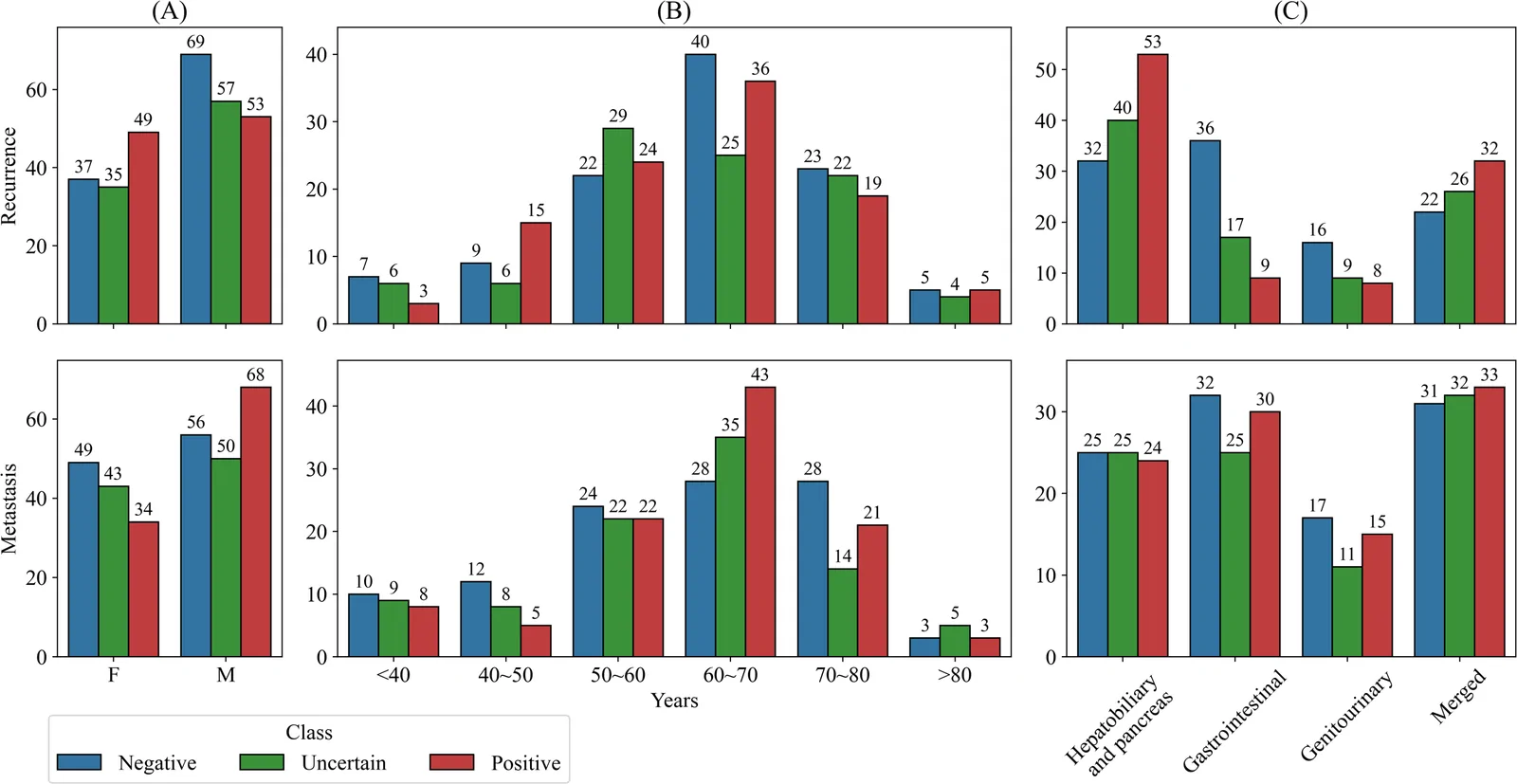
Distribution of predicted recurrence and metastasis labels for 288,076 postoperative computed tomography (CT) reports from 86,083 patients at Asan Medical Center (2014‐2021) across demographic and clinical subgroups: (A) sex; (B) age; (C) cancer type. Merged: all cancer types with a prevalence of <10% of reports, including patients whose cancer type could not be assigned (“missing”).

Table 1 specifies the number of cases stratified by patient gender, age group, cancer type, and their corresponding subcategories, and Figure 3 summarizes the distributions shown in Table 1 into subcategories. Male patients accounted for 15% more cases than female patients, and the number of reports for men was 20% higher. The most common age group was patients aged 60 years to 69 years, followed by those aged 50 years to 59 years. Notably, the total number of counts across age groups exceeded those of gender and cancer type categories, as individuals could contribute to different age groups over time due to aging. Regarding cancer types, the highest frequencies were observed in the following order: gastrointestinal, hepatobiliary and pancreas, and genitourinary. The primary cancer type could not be assigned from the surgical registry for 2283 patients (8028 reports) who are shown as “Missing” in Table 1.

**Table 1.** Number of postoperative computed tomography (CT) reports classified by predicted recurrence and metastasis status (negative, uncertain, and positive), stratified by patient sex, age group, and cancer type for 86,083 patients with cancer who underwent surgery and postoperative CT imaging at Asan Medical Center, Seoul, Republic of Korea (2014‐2021).

Category	Information, n (%)		Prediction, n (%)^a^					
	Patients (n=86,083)	Reports (n=288,076)	Recurrence			Metastasis		
			Negative	Uncertain	Positive	Negative	Uncertain	Positive
Sex								
Female	36,692 (42.6)	114,193 (39.6)	110,550 (96.8)	1455 (1.3)	2188 (1.9)	94,485 (82.7)	8335 (7.3)	11,373 (10)
Male	49,391 (57.4)	173,883 (60.4)	167,800 (96.5)	2552 (1.5)	3531 (2)	147,293 (84.7)	11,587 (6.7)	15,003 (8.6)
Total	—^b^	—	278,350 (96.6)	4007 (1.4)	5719 (2)	241,778 (83.9)	19,922 (6.9)	26,376 (9.2)
Age group (years)^c^								
<40	5466 (6)	15,894 (5.5)	15,444 (97.2)	161 (1)	289 (1.8)	13,142 (82.7)	1077 (6.8)	1675 (10.5)
40‐50	12,353 (13.6)	36,553 (12.7)	35,595 (97.4)	343 (0.9)	615 (1.7)	30,457 (83.3)	2291 (6.3)	3805 (10.4)
50‐60	24,337 (26.7)	78,965 (27.4)	76,432 (96.8)	1028 (1.3)	1505 (1.9)	66,543 (84.3)	5062 (6.4)	7360 (9.3)
60‐70	28,164 (30.9)	92,483 (32.1)	89,241 (96.5)	1393 (1.5)	1849 (2)	78,046 (84.4)	6538 (7.1)	7899 (8.5)
70‐80	17,600 (19.3)	55,119 (19.1)	52,975 (96.1)	919 (1.7)	1225 (2.2)	46,222 (83.9)	4131 (7.5)	4766 (8.6)
≥80	3111 (3.4)	9062 (3.1)	8663 (95.6)	163 (1.8)	236 (2.6)	7368 (81.3)	823 (9.1)	871 (9.6)
Total	91,031 (100)^c^	—	278,350 (96.6)	4007 (1.4)	5719 (2.0)	241,778 (83.9)	19,922 (6.9)	26,376 (9.2)
Cancer type								
Hepatobiliary and pancreas	14,715 (17.1)	77,393 (26.9)	73,077 (94.4)	1780 (2.3)	2536 (3.3)	65,892 (85.1)	4872 (6.3)	6629 (8.6)
Gastrointestinal	28,516 (33.1)	87,075 (30.2)	85,498 (98.2)	726 (0.8)	851 (1)	72,677 (83.5)	5911 (6.8)	8487 (9.7)
Thoracic	6257 (7.3)	16,799 (5.8)	16,388 (97.6)	208 (1.2)	203 (1.2)	13,101 (78)	1832 (10.9)	1866 (11.1)
Breast	6245 (7.3)	12,919 (4.5)	12,691 (98.2)	85 (0.7)	143 (1.1)	10,071 (78)	982 (7.6)	1866 (14.4)
Gynecologic	4535 (5.3)	14,780 (5.1)	14,166 (95.8)	187 (1.3)	427 (2.9)	11,823 (80)	1500 (10.1)	1457 (9.9)
Genitourinary	12,959 (15.1)	39,689 (13.8)	38,636 (97.3)	454 (1.1)	599 (1.5)	34,541 (87)	2158 (5.4)	2990 (7.5)
Neurologic	1498 (1.7)	3855 (1.3)	3745 (97.1)	39 (1)	71 (1.8)	3747 (97.2)	64 (1.7)	44 (1.1)
Hematologic	1442 (1.7)	7682 (2.7)	7524 (97.9)	70 (0.9)	88 (1.1)	7436 (96.8)	141 (1.8)	105 (1.4)
Head and neck	1799 (2.1)	4414 (1.5)	4130 (93.6)	102 (2.3)	182 (4.1)	3493 (79.1)	596 (13.5)	325 (7.4)
Endocrine	3121 (3.6)	5639 (2)	5540 (98.2)	49 (0.9)	50 (0.9)	5078 (90.1)	347 (6.2)	214 (3.8)
Others	2713 (3.2)	9803 (3.4)	9396 (95.8)	161 (1.6)	246 (2.5)	7579 (77.3)	1003 (10.2)	1221 (12.5)
Missing	2283 (2.7)	8028 (2.8)	7559 (94.2)	146 (1.8)	323 (4)	6340 (79)	516 (6.4)	1172 (14.6)
Total	—	—	278,350 (96.6)	4007 (1.4)	5719 (2)	241,778 (83.9)	19,922 (6.9)	26,376 (9.2)

a All classes were classified by the final deep learning (DL) model trained under the human-in-the-loop (HL) semisupervised framework.

b Same as the overall sample total.

c The patient counts in the age group block sum to 91,031, exceeding the unique patient total of 86,083, because a patient could contribute to more than one age bracket over the 2014‐2021 study period as they aged.

### Application Performance

Table 2 shows the accuracy, *F*_1_-score (in the multiclass, *F*_1_-score denotes the macro *F*_1_-score), and weighted *F*_1_-score of both the clinicians and our framework regarding recurrence and metastasis under 3 classification settings (multiclass, positive vs others, and positive vs negative). Human metrics denote the consistency observed across revisions 1 and 2, whereas DL accuracy denotes its concordance with the clinician’s final revision 3. Table S2 in Multimedia Appendix 1 specifies the detailed numbers of each class collected during the 2 revision stages (see the Description of Revisions 1 and 2 section in Multimedia Appendix 1), and the MMD test results are shown in Table S4 in the Consistency of Revision 3’s Distribution section in Multimedia Appendix 1.

**Table 2.** Classification performance of the rule-based algorithm (RA), deep learning (DL) framework, and human clinician benchmark across 3 classification settings for postoperative recurrence and metastasis prediction.

Types, target, and metric	Intrarater	Concordance	
	Human^a^	DL^b^	RA
Multiclass (positive, negative, uncertain)			
Recurrence			
Accuracy	0.9688	0.9733	0.6800
Macro *F*_1_-score	0.7287	0.9731	0.6809
Weighted *F*_1_-score	0.9713	0.9736	0.6797
Metastasis			
Accuracy	0.9380	0.9500	0.6667
Macro *F*_1_-score	0.8059	0.9497	0.6642
Weighted *F*_1_-score	0.9458	0.9503	0.6617
Binary conservative (positive vs others; uncertain grouped with negative)			
Recurrence			
Accuracy	0.9760	0.9933	0.7500
Positive-class *F*_1_-score	0.9857	0.9901	0.6193
Weighted *F*_1_-score	0.9762	0.9933	0.7477
Metastasis			
Accuracy	0.9545	0.9667	0.7733
Positive-class *F*_1_-score	0.9713	0.9505	0.5952
Weighted *F*_1_-score	0.9564	0.9666	0.7585
Binary exclusive (positive vs negative; uncertain cases excluded)			
Recurrence			
Accuracy	0.9942	1.0000	0.7435
Positive-class *F*_1_-score	0.9971	1.0000	0.7135
Weighted *F*_1_-score	0.9942	1.0000	0.7402
Metastasis			
Accuracy	0.9746	0.9898	0.7175
Positive-class *F*_1_-score	0.9867	0.9897	0.6667
Weighted *F*_1_-score	0.9782	0.9898	0.7105

a Intrarater consistency between revisions 1 and 2.

b Concordance with clinician-finalized labels from revision 3 for the 3 classes.

As detailed in Table 2, the framework demonstrated higher concordance with the clinician-finalized gold standard than human intrarater consistency across all evaluated settings; however, because both were assessed against the same clinician-derived labels, this difference reflects closer agreement with the consensus standard rather than superiority over independent clinical judgment. For recurrence classification, the framework achieved an accuracy of 0.9733 in the multiclass task, 0.9933 in the positive vs others setting, and 1.0000 in the positive vs negative setting, whereas the clinicians recorded 0.9688, 0.9760, and 0.9942, respectively. In addition, the framework’s accuracy reached 0.9500 for the multiclass setting, 0.9667 for the positive vs others setting, and 0.9898 for the positive vs negative setting, achieving numerically higher concordance accuracies of 0.9380, 0.9545, and 0.9746, respectively, in the metastasis classification.

**Figure 4.**
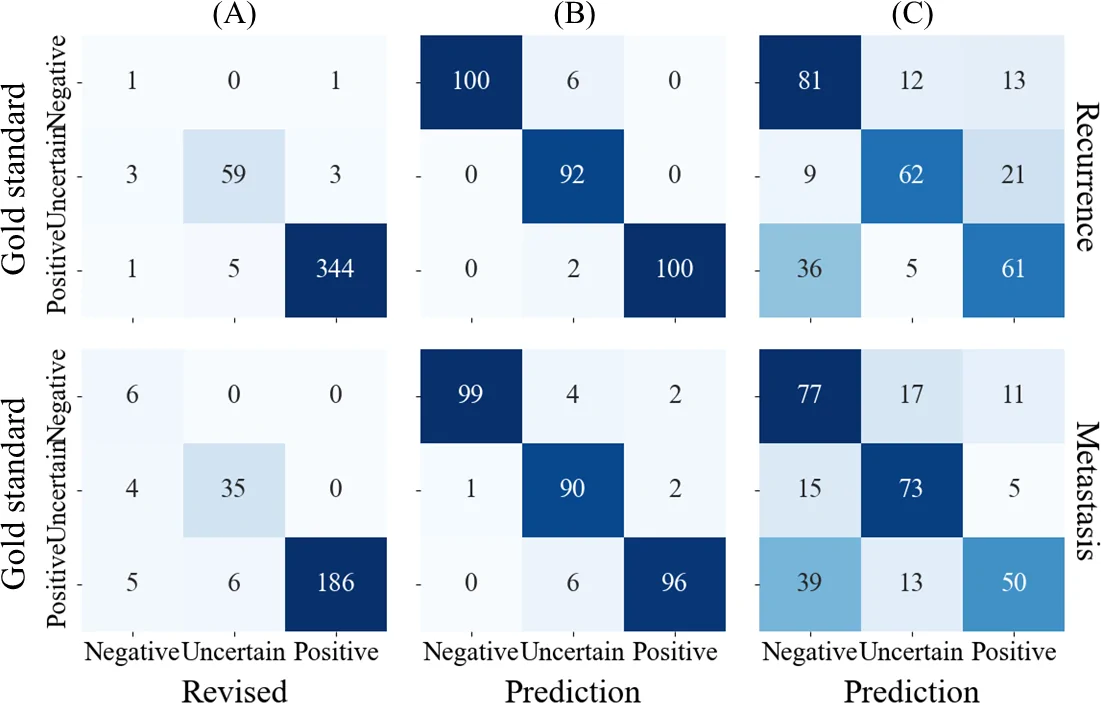
Confusion matrices for multiclass classification (positive, negative, and uncertain) of recurrence and metastasis under the real-world evaluation setting (revision 3), comparing (A) human intrarater consistency, (B) deep learning model predictions, and (C) rule-based algorithm predictions against the gold standard.

**Figure 5.**
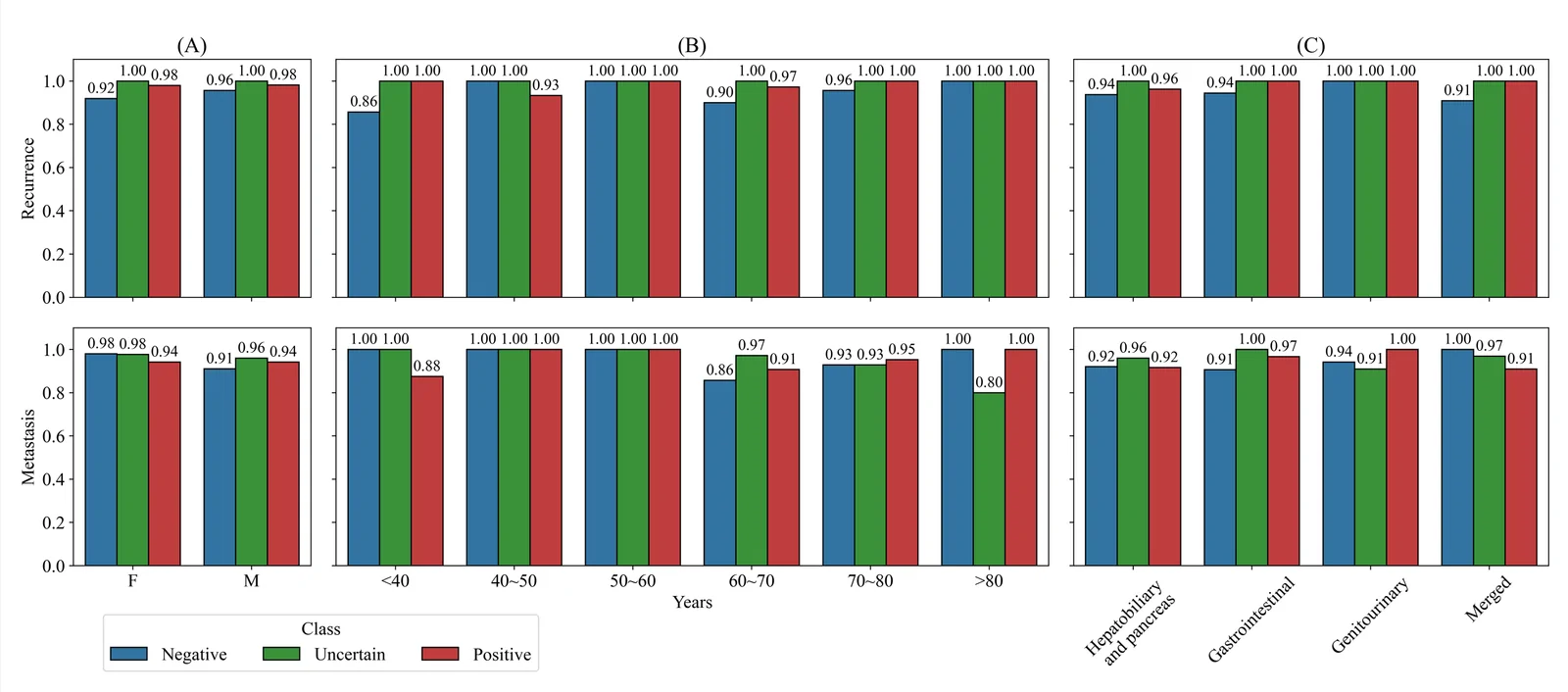
Per-class prediction accuracy of the deep learning model across demographic and clinical subgroups, corresponding to the subgroup distributions shown in Figure 3: (A) sex; (B) age; and (C) cancer type. Merged: all cancer types with a prevalence of <10% of reports, including patients whose cancer type could not be assigned (“missing”).

Figure 4 presents the corresponding confusion matrices for the multiclass setting, confirming that the DL model achieved high classification accuracy across all 3 classes within the revision 3 evaluation set’s 300 samples per target, whereas the RA showed notable misclassification particularly in the positive and uncertain categories. Figure 5 further illustrates the per-class prediction accuracy across demographic and clinical subgroups, demonstrating that the framework maintains consistently high accuracy across sex, age group, and cancer type categories for both recurrence and metastasis. For cancer type subgroups with small sample sizes, underrepresented categories were merged prior to analysis; however, because individual class counts remained limited even after merging, per-case subgroup analysis was not performed due to high performance variability.

### Model Comparison

Table 3 shows that the model comparison performance depends on the evaluation situation. The performance evaluation demonstrated that the RA serves as a competitive baseline, frequently outperforming several sophisticated DL models. Under simulated real-world conditions, the RA achieved higher accuracy than models such as Multilingual-E5 and BlueBERT, particularly in binary metastasis classification. Notably, in the multiclass metastasis classification under the simulation scenario, half of the tested models failed to surpass the RA baseline. Furthermore, only 2 medically pretrained models achieved higher accuracy than the RA in the binary metastasis task. In the recurrence classification, PubMedBERT emerged as the most accurate model across all comparison settings. It outperformed other models in simulated recurrence classification by a margin exceeding 1.5%. During cross-validation under the simulation scenario, PubMedBERT reached average recurrence accuracies of 92.58% in the multiclass and 96.33% in the binary classification. Regarding metastasis classification, BlueBERT achieved the highest accuracy in the multiclass task, demonstrating an improvement of >2% over the next-best model in the naive setting. However, this improvement diminished to <1% under simulated conditions, where BlueBERT reached 89.49%. In the simulated binary metastasis task, BlueBERT’s accuracy was 0.3% lower than that of the RA. By contrast, MedEmbed demonstrated superior reliability for metastasis binary classification under real-world simulation, achieving a final accuracy of 93.25%.

**Table 3.** Cross-validated (10-fold cross-validation) classification performance of 9 language models (LMs) and the rule-based algorithm (RA) under naive and simulated real-world class distributions for postoperative recurrence and metastasis prediction.

Type, target, and model	Multiclass^a^, mean (SD)			Binary class^b^, mean (SD)		
	Accuracy	Macro *F*_1_-score	Weighted *F*_1_-score	Accuracy	Macro *F*_1_-score	Weighted *F*_1_-score
Naive performance						
Recurrence						
RA	0.5225 (0.00)	0.3431 (0.00)	0.4799 (0.00)	0.7197 (0.00)	0.4185 (0.00)	0.6024 (0.00)
Multilingual	0.5099 (0.03)	0.5344 (0.04)	0.4783 (0.04)	0.5637 (0.04)	0.5422 (0.01)	0.5702 (0.05)
BioSimCSE	0.5974 (0.05)	0.5881 (0.05)	0.5900 (0.06)	0.7202 (0.05)	0.6442 (0.04)	0.7334 (0.05)
BioClinical	0.5631 (0.04)	0.5609 (0.04)	0.5514 (0.05)	0.6957 (0.05)	0.6124 (0.04)	0.7099 (0.05)
ClinicalBert	0.5517 (0.04)	0.5610 (0.05)	0.5224 (0.06)	0.6547 (0.05)	0.6044 (0.03)	0.6676 (0.05)
MedEmbed	0.5247 (0.02)	0.5242 (0.02)	0.4932 (0.03)	0.7645 (0.06)	0.6808 (0.05)	0.7749 (0.06)
BiomedBert	0.6087 (0.07)	0.6054 (0.06)	0.6018 (0.07)	0.7145 (0.05)	0.6337 (0.04)	0.7281 (0.05)
BlueBert	0.5559 (0.04)	0.5713 (0.03)	0.5272 (0.05)	0.6424 (0.05)	0.5994 (0.03)	0.6537 (0.06)
PubMedBert	0.6888 (0.07)	0.6748 (0.07)	0.6957 (0.08)	0.8460 (0.06)	0.7633 (0.06)	0.8505 (0.06)
Metastasis						
RA	0.5132 (0.00)	0.4170 (0.00)	0.4583 (0.00)	0.6812 (0.00)	0.4351 (0.00)	0.5860 (0.00)
Multilingual	0.5155 (0.04)	0.5033 (0.05)	0.4781 (0.06)	0.5899 (0.06)	0.5776 (0.03)	0.5915 (0.07)
BioSimCSE	0.5313 (0.05)	0.5174 (0.06)	0.5016 (0.06)	0.6193 (0.07)	0.5951 (0.04)	0.6230 (0.09)
BioClinical	0.5417 (0.05)	0.5276 (0.05)	0.5174 (0.06)	0.6402 (0.06)	0.6080 (0.04)	0.6481 (0.06)
ClinicalBert	0.5177 (0.04)	0.4977 (0.05)	0.4761 (0.05)	0.6288 (0.09)	0.6068 (0.05)	0.6310 (0.10)
MedEmbed	0.5304 (0.04)	0.5068 (0.05)	0.4782 (0.06)	0.7187 (0.06)	0.6692 (0.04)	0.7284 (0.06)
BiomedBert	0.5160 (0.02)	0.5014 (0.03)	0.4881 (0.02)	0.6255 (0.07)	0.5927 (0.05)	0.6329 (0.07)
BlueBert	0.5634 (0.03)	0.5546 (0.03)	0.5296 (0.03)	0.6698 (0.03)	0.6323 (0.02)	0.6798 (0.03)
PubMedBert	0.5197 (0.07)	0.4955 (0.08)	0.4833 (0.09)	0.6897 (0.11)	0.6447 (0.06)	0.6934 (0.13)
Simulated performance						
Recurrence						
RA	0.8863 (0.00)	0.5700 (0.00)	0.8555 (0.00)	0.9336 (0.00)	0.6127 (0.00)	0.9057 (0.00)
Multilingual	0.8837 (0.01)	0.6426 (0.02)	0.8750 (0.01)	0.8968 (0.01)	0.5417 (0.01)	0.9152 (0.01)
BioSimCSE	0.9046 (0.01)	0.7317 (0.04)	0.8983 (0.02)	0.9340 (0.01)	0.6452 (0.04)	0.9424 (0.01)
BioClinical	0.8965 (0.01)	0.7028 (0.03)	0.8870 (0.01)	0.9281 (0.01)	0.6131 (0.04)	0.9373 (0.01)
ClinicalBert	0.8939 (0.01)	0.6811 (0.03)	0.8832 (0.02)	0.9185 (0.01)	0.6056 (0.03)	0.9314 (0.01)
MedEmbed	0.8873 (0.00)	0.6812 (0.02)	0.8653 (0.01)	0.9441 (0.01)	0.6795 (0.05)	0.9501 (0.01)
BiomedBert	0.9068 (0.02)	0.7325 (0.05)	0.9011 (0.02)	0.9321 (0.01)	0.6322 (0.04)	0.9406 (0.01)
BlueBert	0.8949 (0.01)	0.681 (0.03)	0.8855 (0.01)	0.9157 (0.01)	0.6009 (0.03)	0.9295 (0.01)
PubMedBert	0.9258 (0.02)	0.8094 (0.05)	0.9200 (0.02)	0.9633 (0.02)	0.7613 (0.06)	0.9656 (0.01)
Metastasis						
RA	0.8847 (0.00)	0.5709 (0.00)	0.8580 (0.00)	0.9244 (0.00)	0.6237 (0.00)	0.9017 (0.00)
Multilingual	0.8834 (0.01)	0.6357 (0.04)	0.8763 (0.01)	0.9018 (0.01)	0.5745 (0.03)	0.9183 (0.01)
BioSimCSE	0.8871 (0.01)	0.6601 (0.04)	0.8819 (0.01)	0.9089 (0.02)	0.5920 (0.04)	0.9234 (0.01)
BioClinical	0.8895 (0.01)	0.6758 (0.04)	0.8857 (0.01)	0.9137 (0.01)	0.6045 (0.03)	0.9270 (0.01)
ClinicalBert	0.8841 (0.01)	0.6450 (0.04)	0.8745 (0.01)	0.9112 (0.02)	0.6043 (0.05)	0.9255 (0.02)
MedEmbed	0.8869 (0.01)	0.6507 (0.04)	0.8689 (0.01)	0.9325 (0.01)	0.6660 (0.04)	0.9415 (0.01)
BiomedBert	0.8835 (0.01)	0.6554 (0.02)	0.8777 (0.00)	0.9104 (0.02)	0.5898 (0.04)	0.9242 (0.01)
BlueBert	0.8949 (0.01)	0.6717 (0.03)	0.8865 (0.01)	0.9210 (0.01)	0.6306 (0.02)	0.9328 (0.01)
PubMedBert	0.8844 (0.02)	0.6656 (0.07)	0.8738 (0.02)	0.9257 (0.03)	0.6419 (0.06)	0.9361 (0.02)

a Includes positive, negative, and uncertain categories.

b Positive vs others (negative and uncertain combined).

### Preprocessing Analysis

Table 4 presents consecutive postoperative CT reports from a representative patient, illustrating the effect of preprocessing on report content. As shown in Table 4, the majority of reports contained no keyword-matched sentences and were therefore designated as presumed negatives; notably, a subset of the keyword-matched reports yielded identical preprocessed text, constituting duplicate entries prior to deduplication. After exact-match deduplication, the final unique reports counts were 17,846 for recurrence and 63,766 for metastasis. Inspection of the raw text for these excluded reports revealed that their content was clinically unrelated to recurrence or metastasis, consisting instead of descriptions such as adrenal adenoma findings, surgical records, and postoperative complications. In the manual audit of 300 randomly selected keyword-excluded reports, no report was adjudicated as definite radiologic recurrence or metastasis despite the absence of the target keywords, corresponding to an estimated definite missed positive rate of 0% in the reviewed sample. The 95% Clopper-Pearson exact CI for the true missed positive rate was 0%-1.22%, confirming that definite missed positives were unlikely to exceed 1.22% of the presumed-negative pool at the 95% CI. A small number of reports (approximately 2‐3 of 300, or 0.5%‐0.7%) contained indirect or ambiguous nonstandard expressions such as “viable lesion” or “viable tumor.”

**Table 4.** Preprocessing examples of consecutive postoperative computed tomography (CT) reports from a representative patient.

Inspection date	Raw text^a^	Preprocessed text^b^	Recurrence^c^	Metastasis^c^
March 15, 2021	Two 1.5 cm enhancing nodules in both adrenal glands. - absolute washout ratio >60% -- >Adrenal adenoma, most likely. 2. No change of a small indeterminate lymph node around the GE^e^ junction. 3. Two tiny low attenuating lesions in the liver S8 and S7, too small to characterize.	—^d^	—	—
June 2, 2021	Two 1.5 cm enhancing nodules in both adrenal glands. - absolute washout ratio >60% -- >Adrenal adenoma, most likely.	—	—	—
August 20, 2021	No evidence of distant metastasis in abdomen and pelvic cavity. 2. No change of two nodular enhancing lesions in both adrenal glands. - adrenal adenoma, more likely.	No evidence of distant metastasis in abdomen and pelvic cavity. 2. No change of two nodular enhancing lesions in both adrenal glands. adrenal adenoma, more likely.	—	['1. No evidence of distant metastasis in abdomen and pelvic cavity.']
August 20, 2021	Ivor Lewis op for Esophageal cancer. RUL/RLL^f^ segmentectomy for lung cancer. No postop complication or other lesion.	—	—	—
October 8, 2021	Multifocal linear atelectasis in right lung with small pleural effusion. No postop complication in the mediastinum.	—	—	—
October 8, 2021	No evidence of metastasis in abdomen and pelvic cavity.	No evidence of metastasis in abdomen and pelvic cavity.	—	['No evidence of metastasis in abdomen and pelvic cavity.']
October 18, 2021	Slightly increased metabolism and extent of mild hypermetabolic lymph node in right upper paratracheal area, compared with 2021-02-09 PET^g^/CT: Reactive change >Metastatic lymph node REC^h^) Chest CT close follow-up or EBUS^i^ correlation 2) No significant change of probable reactive lymph nodes in both lower paratracheal, subaortic, subcarinal, both pulmonary hilar and interlobar area 3) No evidence of tumor recurrence in esophagus and right lung operative bed 4) No significant change of biopsy proven benign nodule in left lobe of thyroid gland4 5) Otherwise, no significant hypermetabolic lesion	Slightly increased metabolism and extent of mild hypermetabolic lymph node in right upper paratracheal area, compared with 2021-02-09 PET/CT: Reactive change >Metastatic lymph node REC) Chest CT close follow-up or EBUS correlation 2) No significant change of probable reactive lymph nodes in both lower paratracheal, subaortic, subcarinal, both pulmonary hilar and interlobar area 3) No evidence of tumor recurrence in esophagus and right lung operative bed 4) No significant change of biopsy proven benign nodule in left lobe of thyroid gland4 5) Otherwise, no significant hypermetabolic lesion	[' No evidence of tumor recurrence in esophagus and right lung operative bed’]	['1) Slightly increased metabolism and extent of mild hypermetabolic lymph node in right upper paratracheal area, compared with 2021-02-09 PET/CT: \nReactive change >Metastatic lymph node\nREC) Chest CT close follow-up or EBUS correlation’]
February 24, 2022	Multifocal linear atelectasis in right lung with small pleural effusion. No postop complication in the mediastinum. 2. No change of small LNs^j^ in both upper and lower paratracheal areas. --- >R/O^k^ reactive LNs vs metastasis.	Multifocal linear atelectasis in right lung with small pleural effusion. No postop complication in the mediastinum. 2. No change of small LNs in both upper and lower paratracheal areas. Rule Out reactive LNs vs metastasis.	—	['No change of small LNs in both upper and lower paratracheal areas.\nRule Out reactive LNs vs metastasis. \n’]
February 24, 2022	No evidence of metastasis in abdomen and pelvic cavity.	No evidence of metastasis in abdomen and pelvic cavity.	—	['No evidence of metastasis in abdomen and pelvic cavity.']

a Original radiology report impression.

b Result after cleaning and keyword-based filtering.

c Only the sentences retained for each target after preprocessing, illustrating that most reports are either entirely excluded or reduced to 1 or 2 short sentences.

d No keyword-matched sentences were identified, and the corresponding report was dropped from the target dataset.

e GE: gastroesophageal.

f RUL/RLL: right upper lobe/right lower lobe.

g PET: positron emission tomography.

h REC: recommendation.

i EBUS: endobronchial ultrasound.

j LNs: lymph nodes.

k R/O: rule out.

Furthermore, reports that passed keyword filtering were reduced to 1 or 2 short sentences per target, demonstrating that the preprocessing pipeline fragmented each report into brief, context-independent text segments that eliminated patient-level linguistic cues and temporal continuity across sequential reports. To further validate the weak-negative designation, a board-certified radiologist reviewed all weak-negative reports identified by the preprocessing pipeline. For recurrence, 1 of 486 weak-negative reports was adjudicated as positive, yielding a 95% CI for the true mislabeling rate of 0.005%-1.143%, indicating that the mislabeling rate for recurrence was extremely low, with an upper bound of 1.1% at the 95% CI. For metastasis, 19 of 953 weak-negative reports were adjudicated as positive, with an additional 4 designated as uncertain; the corresponding 95% CI was 1.200%-3.101% when considering definite positives only and 1.531%-3.607% under the conservative assumption that uncertain cases represented mislabeled positives. These results collectively confirmed that the weak-negative samples constituted a statistically reliable approximation of true negatives, with mislabeling rates bounded at <3.6% across both tasks at the 95% CI. All raw reports underwent preprocessing, and the resulting efficiency is illustrated in the Preprocessing Efficiency and Unused Negative Samples sections in Multimedia Appendix 1.

### Model Interpretation

**Figure 6.**
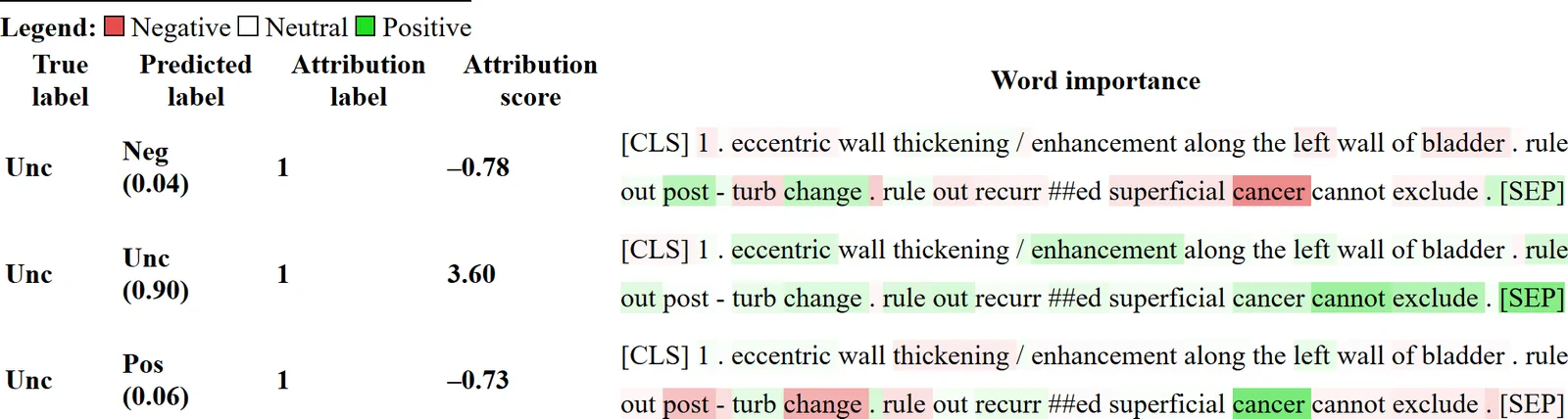
Token-level integrated gradients attribution scores for a representative uncertain recurrence report (revision 3), with each row presenting the same report with predicted labels of negative (neg; 0.04), uncertain (unc; 0.90), and positive (pos; 0.06), respectively.

Figure 6 shows an interpretation of the model’s predictions for an uncertain recurrence report. As shown in the figure, when making predictions, the model relied not only on the tokens “recurr,” “##ed,” and “cancer” but also on nearby related tokens. The phrase “cannot exclude” received the highest attribution score (3.60) in the uncertain prediction, demonstrating that the model appropriately captured hedging expressions as indicators of diagnostic uncertainty.

## Discussion

### Principal Findings

Our study demonstrated that a semisupervised learning, HL framework is feasible for large-scale automated extraction of postoperative cancer recurrence and metastasis from unstructured CT radiology reports, screening and classifying all 288,076 reports in the corpus. Our framework achieved concordance rates of 97.33% for recurrence and 95.00% for metastasis against clinician-finalized gold standard labels (revision 3), with corresponding human intrarater consistency of 96.88% and 93.80%, respectively. These figures demonstrate the degree to which the model reproduces the consensus labeling standard established through iterative clinician review; because both the model and the human benchmark were evaluated against the same clinician-derived gold standard, the reported advantage does not constitute evidence of superiority over independent clinical judgment. In practical terms, clinician screening of 1000 samples required more than 1 week within our annotation workflow—encompassing case review and adjudication of ambiguous cases—whereas the automated framework processed the equivalent volume in under 10 minutes while maintaining high concordance with clinician labels. Although this comparison reflects the specific conditions of our annotation process and may not directly generalize to other clinical review settings, it illustrates the scalability advantage of the proposed approach for labeling at the scale of 288,076 screened reports. After exact-match deduplication, the models were trained and evaluated on 17,846 unique reports for recurrence and 63,766 for metastasis; deduplication was performed separately for each target to prevent leakage introduced by report-level splitting. This performance was achieved through only 3 revision cycles, each involving approximately 2000 samples (less than 1% of the 288,076 total reports), underscoring the annotation efficiency of the HL design. The framework captured diagnostic uncertainty in 1.4% of recurrence and 6.9% of metastasis cases, addressing a critical gap in existing binary classification approaches [12,27].

RA served as a competitive baseline, outperforming several DL models under simulated real-world conditions, particularly for binary metastasis classification. This finding underscores the value of medically grounded heuristics within hybrid systems [1,8]. Among DL models, PubMedBERT achieved the highest accuracy for recurrence (92.58% under simulation, multiclass classification), while MedEmbed demonstrated superior reliability for binary metastasis classification (93.25%). The use of domain-specific BERT models aligns with findings that biomedical language representation pretraining significantly improves text mining performance [13,14], and recent investigations support the task-specific advantages of PubMedBERT and MedEmbed for recurrence and metastasis classification, respectively. The level of inconsistency between weak and gold labels is reflected in the intrarater disagreement rate observed across clinician review cycles (3.12% for recurrence and 6.20% for metastasis), which serves as an empirical estimate of the annotation noise present in the labeling process and an upper bound on achievable model performance.

### Comparison With Prior Work

The primary contribution of this work lies in the integration of established components—rule-based prelabeling, BERT-based LMs, embedding-based clustering, and iterative HL refinement—within a clinically grounded workflow that explicitly models diagnostic uncertainty as a prospectively defined, independent class. Prior studies on automated extraction of oncologic outcomes from radiology reports have generally simplified borderline impressions into binary categories or excluded ambiguous cases from training and evaluation entirely [10,12,27]. Related previous studies have documented substantial challenges with extracting structured information from radiology reports, including linguistic ambiguity, negations, and diagnostic uncertainty [1,7], and institutional and stylistic variation has been identified as a major obstacle to automated extraction systems [1]. In contrast, our 3-class framework captures the clinical reality in which radiologists frequently encounter findings that are suspicious but not definitive [9]. Studies on automated extraction from radiology reports have emphasized that accounting for diagnostic uncertainty is essential to maintain clinical utility [8,11].

Our presumed labeling hybrid strategy combining RA, UMAP-DBSCAN clustering, and semisupervised classification was designed to operate as a sequential pipeline in which each stage builds upon the preceding step. Nonetheless, the practical necessity of the weak-negative clustering step was evident from the annotation burden it prevented: had this step been omitted, clinician review of the negative category would have increased by approximately 20% without a corresponding improvement in label quality for that class. Previous work on semisupervised text topic modeling provided a conceptual foundation [17], although our adaptation required domain-specific modifications for medical radiology reports. UMAP-DBSCAN clustering for presumed negative identification reduced clinician review burden while maintaining high accuracy, with MMD confirming the distributional integrity of the sampled data [25]. The need for quantifiable and objective textual approaches in radiology reporting has been emphasized [2], and our framework addresses this through domain-specific LMs combined with presumed-labeling strategies, consistent with recent work on clinically informed semisupervised learning from electronic health records [28].

The RA assigned “rule out” expressions uniformly as uncertain, regardless of whether such language reflected a diagnostic finding or a clinical indication for the examination. This semantic overlap represents a known limitation of keyword-based prelabeling in radiology NLP, as “rule out” frequently appears in the indication section of a report rather than the impression or findings sections [1,8]. Because the preprocessing pipeline did not separate indication from impression, some “rule out” assignments may have been misclassified at the RA stage. However, this ambiguity was mitigated by training the DL models exclusively on clinician-reviewed gold standard labels, including uncertain cases verified through the HL revision cycles. As a result, the impact of this initial labeling inconsistency on final model performance was limited, as evidenced by the large performance gap between the RA and DL models on the Uncertain class in the confusion matrices (Figure 4).

The substantial performance differences between naive and simulation evaluations emphasize the importance of realistic assessment conditions. Previous research has noted that weakly supervised DL in radiology requires careful evaluation under conditions reflecting real-world class distributions [3,5,29]. MedEmbed’s multiclass metastasis accuracy improved from 53.04% under naive conditions to 88.69% under simulated real-world distributions, demonstrating that model selection based solely on balanced test sets may not identify optimal models for clinical deployment scenarios with pronounced class imbalance.

The successful processing of 288,076 radiology reports across 11 cancer types compares favorably with prior large-scale studies on automated metastasis detection from pathology reports [12] and extends the scope of clinical NLP to postoperative CT surveillance across a broad oncologic case mix. Traditional cancer registries rely heavily on manual abstraction, limiting scalability and timeliness [12]. Previous studies have documented the challenges of incorporating cancer recurrence events into population-based registries [6], and scoping reviews have noted that NLP of radiology reports in oncology remains an evolving field [10]. Within the institutional setting of this study, our framework has the potential to support timely construction of comprehensive surveillance databases, facilitating population-level quality monitoring and outcome research [30].

Recent work emphasized that clinicians must participate in the development of multimodal artificial intelligence (AI) to ensure clinical relevance and trustworthiness [31]. The interpretability analysis via IG [26] provided transparency essential for clinical acceptance and demonstrated that the model attends to clinically relevant tokens–such as “cannot exclude” for uncertain–rather than spurious correlations, providing the transparency necessary for clinical acceptance and aligning with this principle.

### Limitations

Several limitations of this study should be acknowledged.

First, all reports were collected from a single institution, which may limit generalizability to other health care settings with different reporting styles and terminology [12]. Recent work has noted that heterogeneity in AI assistance effects across radiologists reflects variations in practice patterns and institutional contexts [26,32], suggesting that multicenter validation is essential. In this study, external validation was not feasible due to the absence of a comparable annotated dataset from another institution at the time of analysis. To mitigate this, performance was reported under 3 complementary evaluation settings (naive, simulation, real world) to approximate the range of conditions likely encountered in practice. External validation across multiple institutions with diverse reporting conventions remains necessary to confirm broader applicability [5,30].

Second, preprocessing relies on keyword filtering (“recur” and “metas”), which may miss cases described using synonymous or indirect language without these specific terms. This design assumes that radiologists explicitly document recurrence or metastasis using these root strings—an assumption clinically justified by institutional documentation standards, where failure to use standard terminology for critical findings has direct patient safety implications. Although previous studies have successfully used keyword-based approaches for oncologic information extraction [8,10], the potential for systematic exclusion of atypical descriptions cannot be fully eliminated. To partially address this concern, we sampled a manual audit of 300 random keyword-excluded reports, in which no report was adjudicated as definite recurrence or metastasis (estimated definite missed-positive rate: 0%, 95% CI 0.00%‐1.22%); qualitative inspection of excluded report content further confirmed clinical unrelatedness to recurrence or metastasis in the reviewed sample (Table 4). However, a small proportion of audited reports (approximately 0.5%‐0.7%) contained indirect or ambiguous expressions such as “viable lesion” or “viable tumor” that could not be confidently adjudicated without longitudinal imaging or clinical correlation, and the audit covered only a subset of the full presumed-negative pool. Although the upper bound of the 95% CI was 1.22%, the large scale of the presumed-negative pool means that even a small missed-positive rate could correspond to a nonnegligible absolute number of cases in practice. In addition, weak-negative samples further confirmed low mislabeling rates, with 95% CIs of 0.005%-1.143% for recurrence and up to 1.531%-3.607% for metastasis under conservative assumptions. Future studies should therefore consider broader lexical matching, context-aware screening, or large language model–based preprocessing to capture such nonstandard expressions without overclassifying clinically ambiguous reports as definite recurrence or metastasis.

Third, the semisupervised pipeline designated reports as weak-negatives through embedding-based clustering without subsequent clinician review in order to minimize annotation burden. Although MMD testing confirmed that these weak-negative samples were distributionally consistent with the broader clinical population, a residual risk of label contamination existed if atypical positive or uncertain cases clustered among negatives. A random sample of weak-negative reports was reviewed, confirming that the automated labeling process was accurate for the reviewed subset. However, a systematic false-negative rate across the full weak-negative pool was not formally established, and this uncertainty may introduce noise into training data for the negative class. Future work should consider establishing a formal baseline error rate through structured sampling of the weak-negative cluster.

Fourth, all annotations were performed by a single attending physician, which introduces the risk of individual annotator bias and limits the reliability of the gold standard as a true consensus label. Although ambiguous cases were adjudicated in consultation with a faculty radiologist, formal interrater reliability was not established through independent double annotation. The impact of this limitation is most pronounced for the uncertain class, where classification criteria are inherently subjective and boundary cases between positive and uncertain are particularly sensitive to individual clinical judgment. The intrarater consistency metrics reported in this study therefore reflect temporal stability of a single annotator’s decisions rather than convergence across multiple independent reviewers. Future studies should incorporate structured multi-annotator protocols with adjudication procedures to establish a more robust gold standard, particularly for the uncertain category.

Fifth, the framework processes each CT report as an independent document without access to a patient’s prior imaging history or longitudinal clinical trajectory. Cancer recurrence and metastasis are inherently longitudinal events in which temporal context, such as interval change from a previous study, is central to radiologist interpretation. The absence of this temporal information may limit the clinical utility of single-report predictions, particularly for cases where the current report alone is ambiguous but the longitudinal trend is diagnostic. Future architectures incorporating sequential report representations or structured temporal features may better approximate the interpretive process of clinical radiologists.

Sixth, exact-match deduplication, which was applied to prevent leakage from report-level splitting, also removes the natural frequency with which common clinical phrases recur, so the deduplicated evaluation set reflects the distribution of distinct phrasings rather than the frequency-weighted distribution of routine reporting. The reported performance should therefore be interpreted accordingly, and prospective evaluation on unfiltered, frequency-weighted report streams is warranted to confirm generalizability to real-world surveillance.

### Future Directions

Several directions merit exploration in future work. First, multicenter prospective validation is the most critical next step to establish generalizability. Transfer learning from institution-specific LMs may enable efficient domain adaptation with minimal additional annotation [13,33], and domain adaptation strategies should explicitly address differences in reporting language and terminology across institutions [5].

Second, incorporating temporal context into the classification pipeline through sequential report modeling or structured longitudinal representations could improve performance on inherently ambiguous single-report cases and better approximate clinical reasoning in postoperative surveillance.

Third, active learning strategies within the HL framework could further reduce annotation burden for rare cancer subtypes, where current subgroup performance is most limited [10,24]. Cancer-specific fine-tuning using targeted data collection may also address the performance gaps observed in breast and gynecologic cancers.

Fourth, integration with hospital information systems via MLOps pipelines would enable continuous model retraining and real-time surveillance, accommodating temporal shifts in imaging technology and reporting standards [34]. Structured HL feedback mechanisms are essential for sustainable deployment.

### Conclusions

This study developed and evaluated a semisupervised, HL framework for classifying postoperative CT radiology reports into 3 clinically meaningful categories—positive, negative, and uncertain—for cancer recurrence and metastasis. The framework achieved concordance with clinician-finalized labels at rates of 97.33% for recurrence and 95.00% for metastasis in multiclass classification, matching the human intrarater consistency observed in the same annotation process while requiring clinician review of <1% of the total 288,076 screened and classified reports across 3 revision cycles; after exact-match deduplication, models were trained and evaluated on 17,846 and 63,766 unique reports for recurrence and metastasis, respectively. Explicit modeling of diagnostic uncertainty captured a clinically nonnegligible proportion of cases that would be misassigned in binary classification schemes. These findings demonstrate that the proposed framework can achieve annotation-efficient, clinician-concordant classification of oncologic outcomes within a large-scale, single-institution postoperative CT radiology corpus; generalizability to other institutions and reporting conventions requires prospective multicenter validation.

## Supplementary material

10.2196/92937 Multimedia Appendix 1 Additional methods, descriptions of the training and validation sets, and unused negative samples.

## Abbreviations

AI: artificial intelligence
CT: computed tomography
DL: deep learning
HL: human in the loop
IG: integrated gradients
LM: language model
MMD: maximum mean discrepancy
NLP: natural language processing
RA: rule-based algorithm
