# Supplementary material for: Automated Extraction of Postoperative Cancer Recurrence and Metastasis From Computed Tomography (CT) Reports: Semisupervised Deep Learning Study
Source: JMIR Med Inform. 2026 Sep 8;14:e92937. doi: 10.2196/92937 (PMC13552650; doi:10.2196/92937)
Supplement: Supplementary file 1 [file medinform-v14-e92937-s001.docx]

# Supplementary materials

## Details of preprocessing

To minimize computational expense and improve learning efficiency compared with training on the full raw reports, nontarget textual content was excluded during preprocessing, consistent with previous work.[S1, S2, S3, S4, S5] **Figure 1** in the main manuscript illustrates the preprocessing pipeline and a representative example.

In the first step, commonly used clinical abbreviations in postoperative CT reports are expanded to their full expressions. For instance, “R/O” is converted to “rule out,” and a comprehensive list of abbreviations appearing in the dataset is provided in **Supplementary Table 1***.* During the abbreviation preprocessing, our approach incorporated both medical terminology and common expressions. To ensure robust text standardization, we implemented case-insensitive matching; for instance, all variations of P/O (e.g., P/O, P/o, p/O, p/o) were converted to their corresponding full text, regardless of their original capitalization.

In addition, excessive whitespace and delimiter or arrow characters were subsequently removed to avoid interference with segmentation patterns (e.g., “1)”, “1.”), which may otherwise result in incorrect segment identification. After text cleaning, each report is divided into segments based on these structural patterns. Following segmentation, each segment is searched using regular expressions for recurrence-related “recur” or metastasis-related “metas” keywords. These terms were selected because they broadly encompass the majority of expressions related to postoperative recurrence or metastasis in clinical reporting. Segments containing any of the target keywords are retained, whereas segments without relevant keywords are excluded, as illustrated by the red boxes in the preprocessing blocks in **Figure 1** in the main manuscript. The retained segments are then concatenated into a single text and assigned to either the recurrence or metastasis dataset depending on the detected keyword category.

Supplementary Table 1. Abbreviation list for preprocessing

| Full text | Abbreviation | Full text | Abbreviation |
| --- | --- | --- | --- |
| Associated With | A/W | Rule Out | R/O |
| Consistent With | C/W | Retroperitoneal | R/P |
| Due To | D/T | Status Post | S/P |
| Fluids and Electrolytes | F/E | To Discharge | T/D |
| Follow Up | F/U | Urinalysis | U/A |
| Lymph Node | L/N | Without | W/O |
| Post-Operative | P/O | Work Up | W/U |

## Details of labeling

Considering that no reference-standard annotations were available at the beginning of the study, two labeling strategies were implemented: (1) clinician-derived golden-standard annotations and (2) weak labeling, which is a semi-supervised approach in which all reports are projected through an LM to identify samples estimated as negative and incorporate them as training labels.

Skilled clinicians conducted three independent rounds of review to construct the golden-standard annotations. To reduce unnecessary expert workload, initial drafts were pre-classified using a simple RA [S5], given that a substantial portion of postoperative reports did not contain information relevant to the target outcomes. The temporal process of golden-standard construction is illustrated by the HL schedule in **Figure 1** in the main manuscript. During Revision 1, the review primarily focused on positive cases. In Revision 2, both positive and uncertain cases were reviewed in equal proportions. Instances of text-level discrepancy were documented and are addressed in **Supplementary Table 2**.

A dedicated clinician review for negative cases was not performed in view of the consistently high proportion of negative predictions across the unlabeled reports. Although preprocessing generated presumed negatives, LM-based text embeddings indicated that additional nontarget negative samples were still present. Accordingly, a semi-supervised learning strategy was adopted, combining the pre-labeling algorithm with embedding-based clustering to identify samples consistently assigned to the negative class across methods, designating them as weak negatives.[S6]

The text embedding–clustering procedure is as follows in **Figure 1** in the main manuscript**.** First, each report sample that was prelabeled was embedded using PubMedBERT.[S7] The embeddings were decomposed using UMAP [S8] and clustered by DBSCAN [S9] for the decomposed vectors. Examples of trainable weak negatives are illustrated by the orange circles in the prelabeled weak-negative region located in the upper-right subfigure of **Figure 1** in the main manuscript. The samples within these orange-marked clusters satisfy the required conditions; they are prelabeled negative and belong to only the negative cluster. Direct clustering on high-dimensional embeddings did not yield meaningful separation, as samples aggregated into a single cluster, limiting the identification of weak negatives.

Supplementary Table 2. Training and validation set with revision 1 and 2

|  | Class | Drop duplicates | | | | | | Raw | | | | | |
| --- | --- | --- | --- | --- | --- | --- | --- | --- | --- | --- | --- | --- | --- |
|  |  | Revision 1 | | | Revision 2 | | | Revision 1 | | | Revision 2 | | |
|  |  | Unique | Double  checked | Sum | Unique | Double  checked | Sum | Unique | Double  checked | Sum | Unique | Double  checked | Sum |
| Recurrence | Positive | 460 | 348 | 808 | 162 | 346 | 508 | 465 | 350 | 815 | 163 | 348 | 511 |
|  | Uncertain | 50 | 65 | 115 | 332 | 64 | 396 | 50 | 65 | 115 | 334 | 64 | 398 |
|  | Negative | 162 | 2 | 164 | 84 | 5 | 89 | 170 | 2 | 172 | 86 | 5 | 91 |
|  | Sum | 672 | 415 | 1087 | 578 | 415 | 993 | 685 | 417 | 1102 | 583 | 417 | 1000 |
| Metastasis | Positive | 651 | 194 | 845 | 225 | 183 | 408 | 672 | 197 | 869 | 226 | 186 | 412 |
|  | Uncertain | 12 | 37 | 49 | 345 | 39 | 384 | 12 | 39 | 51 | 346 | 41 | 387 |
|  | Regional | 6 | 5 | 11 | 52 | 6 | 58 | 6 | 5 | 11 | 52 | 6 | 58 |
|  | Negative | 58 | 1 | 59 | 134 | 9 | 143 | 72 | 1 | 73 | 134 | 9 | 143 |
|  | Sum | 727 | 237 | 964 | 756 | 237 | 993 | 762 | 242 | 1004 | 758 | 242 | 1000 |

## Details of text classification

**Supplementary Figure 1** shows an illustration of the three labeling methods. We used three methods to classify each text into the target classes: (1) a pre-labeling RA for weak labeling, (2) an LM classifier, and (3) contrastive learning. The left subfigure in **Supplementary Figure 1** illustrates the resulting pre-labeling procedure. The keyword “rule out” functioned as the primary indicator for identifying uncertain cases; accordingly, any sample containing this term was labeled uncertain. A list of terms with negative meaning was also compiled, and samples in which these negative terms appeared less frequently than the target terms (“metas” or “recur”) were assigned to the positive class. All remaining cases were labeled as negative.

Although these rules did not capture all possible expressions, they provided sufficiently consistent initial labels for use in subsequent clinician review. Weak-negative samples, together with the clinician-reviewed uncertain and positive labels, were used to train the LM classifier. All negative and uncertain patterns are listed in **Supplementary Table 1**. The keyword-based preprocessing and RA, despite their simplicity, rely on medically grounded regular-expression patterns. By focusing on explicit clinical target keywords, this streamlined procedure isolates relevant cases without requiring computationally intensive neural architectures and establishes a robust baseline for subsequent modeling.

The LM classifier, shown in **Supplementary Figure 1**, has a simple two-part structure: (1) a backbone and (2) a head. Following previous studies on medical text representation [S10], domain-specific BERT models were used as backbones. Cross-validation identified MedEmbed BERT [S11] and PubMedBERT [S7] as the best-performing models for metastasis and recurrence classification, respectively. The classifier head generates three outputs corresponding to the three target classes, which are converted to probabilities using a softmax function. Cross-entropy loss is commonly used for such tasks; however, the strong imbalance in the uncertain class, as noted in **Supplementary Table 2**, reduced its effectiveness.[S12] We used focal loss to stabilize learning under severe class imbalance.[S13]

Supplementary Figure 1. Illustration of the three classification methods: (Left) the pre-labeling rule-based algorithm (RA) assigning weak labels based on target keywords and negation patterns; (Middle) the language model (LM) classifier comprising a domain-specific BERT backbone and a three-class softmax output head; and (Right) the contrastive learning module optimizing embedding similarity between same-class (anchor–positive) and different-class (anchor–negative) pairs.

Contrastive learning was applied in Prediction 2, which involved training on the metastasis subset of Revision 1 and generating predictions for the test set. As shown in the Supplementary Materials, uncertain samples constituted only a small fraction of the Revision 1 golden-standard labels. To facilitate the representation of uncertain cases, a hybrid supervised–unsupervised approach was examined. In a pilot experiment for Prediction 1, only the unsupervised component successfully distinguished uncertain cases, whereas supervised learning failed to do so. For the unsupervised component, PubMedBERT was used as the backbone and optimized using the InfoNCE loss,[S14] a standard method in biomedical representation learning. One positive and four negative instances were sampled uniformly with replacement for contrastive pairing. Contrastive learning was effective only when applied to the metastasis subset of Revision 1; in all other settings, supervised learning demonstrated superior performance. Additional methodological details are therefore omitted

## Details of statistical testing

This section outlines the validation procedure employed to assess whether the sampling strategy for the Golden-Standard dataset accurately represents the underlying distribution of the corresponding subpopulation. We posit that distinct classes exhibit unique distributional characteristics and varying sample sizes; indeed, such distributional divergence is essential for the learnability of the classification task. To validate the representativeness of the sampled subset, we mapped all reports into a high-dimensional feature space using a pretrained LM.[S15] This approach is grounded in empirical findings demonstrating that statistical tests on dimensionality-reduced representations are highly effective for detecting dataset shifts.[S16]

Although we initially considered Hotelling's $T^{2}$ test to compare the multivariate means, this approach was deemed unsuitable because large sample sizes can lead to vanishing $p$-values,[S17] and our sample size is smaller than the dimensionality of the embedding vectors.[S18] Consequently, to verify that the distribution of each class in Revision 3 is consistent with that of the corresponding population subset predicted by the model, we employed the maximum mean discrepancy (MMD) test for each class.[S19] This methodology aligns with the core-set approach, ensuring that the selected samples sufficiently cover the geometric manifold of the entire dataset within the feature space.[S20]

MMD is defined as the difference between the mean embeddings of two probability distributions in a reproducing kernel Hilbert space $\mathcal{H}$. It is a nonparametric measure capable of comprehensively capturing differences not only in the mean but also in higher-order moments.[S19] Because the unbiased estimator of standard MMD has a computational complexity of $\mathcal{O}\left( N^{2} \right)$, it incurs prohibitive computational costs and memory constraints during kernel matrix computation when applied to large-scale populations.

To address these limitations, we adopted the linear-time MMD estimator,[S19] which drastically reduces the complexity to $\mathcal{O}\left( N \right)$ by utilizing independent sample pairs rather than processing the entire dataset. In this test, let $\mathcal{X}_{S}$ and $\mathcal{X}_{T}$ denote the source population and the target labeled sample, respectively. We performed a linear approximation by randomly subsampling from the corresponding class in $\mathcal{X}_{S}$ using a batch size $B$, which determines the number of independent pairs used for the approximation. The linear MMD statistic $MMD_{l}^{2}$ is calculated as follows:

$$MMD_{l}^{2}\left( \mathcal{X}_{S},\mathcal{X}_{T} \right)=\frac{1}{B}\sum_{i=1}^{B} h\left( z_{i},z_{i}^{'} \right)$$

Here, the term $h\left( z_{i}, z_{i}^{'} \right)$ serves as an unbiased kernel operator that aggregates intraclass and interclass kernel similarities. This operator is computed using independent samples $x_{S,i}$ and $x_{S,i}^{'}$ drawn from $\mathcal{X}_{S}$ and $x_{T,i}$ and $x_{T,i}^{'}$ drawn from $\mathcal{X}_{T}$. The function $k\left( \cdot, \cdot\right)$ represents the Gaussian radial basis function kernel corresponding to the inner product in $\mathcal{H}$. The kernel operator is explicitly defined as follows:

$$h\left( z_{i},z_{i}^{'} \right)=k\left( x_{S,i},x_{S,i}^{'} \right)+k\left( x_{T,i},x_{T,i}^{'} \right)-k\left( x_{S,i},x_{T,i}^{'} \right)-k\left( x_{S,i}^{'},x_{T,i} \right)$$

To capture nonlinear relationships within the embedding space, the Gaussian radial basis function kernel is defined as $k\left( x,y \right)=e^{-\gamma\left\| x-y \right\|^{2}}$. The kernel bandwidth parameter $\gamma$ was determined using the median heuristic, which sets $\gamma$ to the inverse of the median squared Euclidean distance between data points. This approach optimizes the parameter according to the scale of the distribution.[S19]

## Details of experimental setup

We describe our experiments in three parts: the experimental environment, the model configurations, and the evaluation metrics. All experiments were performed in a conda environment built with Python 3.12 and CUDA 12.8 based on Windows 11, i5-13400F, and RTX3090. For the main libraries, we used PyTorch 2.9.0, PyTorch-Lightning 2.5.5, Transformers [S21] 4.57.1, and captum[S22] 0.8.0. The LMs used as backbones were selected based on prior studies.[S23] Along with recent medical domain models, we also included Multilingual-E5,[S24] which focused on general domain NLP. Performance comparisons between models are summarized in ***Model comparison*** in the main manuscript, and detailed descriptions of each model are provided in **Supplementary Table 3**.

Training was optimized using early stopping with five epochs of patience and a learning rate $lr=1\times{10}^{-5}$. For the clustering-based construction of weak-negative labels, the DBSCAN epsilon was set to 0.1. The balancing parameter $\gamma_{focal}$ of the focal loss was set to 2.0. We also trained the model to converge enough with five early stopping rounds. We performed the $N_{perm}=1000$ permutation for the MMD test, and the other hyperparameter that we do not mention is set up to default. All experiments were run with 42 fixed random seeds to ensure reproducibility.

Two metrics were used to evaluate model performance: accuracy and F1 score. Because our experiments include both multiclass and binary-class classification (positive vs. others), we used Macro-F1 for the multiclass task and F1 considering only positive for the binary task. Because macro F1 treats all classes equally without considering the frequency $n_{c}$ of each class, we additionally report weighted F1 to resolve class imbalance.[S25] TP, TN, FP, and FN denote true positive, true negative, false positive, and false negative, respectively. The equations for each metric are as follows:

| $Accuracy=\frac{TP+TN}{TP+TN+FP+FN}$  ${Precision}_{c} = \frac{{TP}_{c}}{{TP}_{c} + {FP}_{c}}$  ${Recall}_{c} = \frac{{TP}_{c}}{{TP}_{c} + {FN}_{c}}$ | ${F1}_{c}=\frac{2\cdot{Precision}_{c}\cdot{Recall}_{c}}{{Precision}_{c}+ {Recall}_{c}}$  $F1={F1}_{Positive}$  $\mathrm{Macro}-F1 = \frac{1}{C} \sum_{c=1}^{C} {F1}_{c}$  $\mathrm{Weighted}-F1 =\frac{1}{\sum_{i=1}^{C} n_{i}}\sum_{c=1}^{C} n_{c}\cdot{F1}_{c}$ |
| --- | --- |

Supplementary Table 3. The list of DL backbones

| Name | Parameter | Huggingface repo id |
| --- | --- | --- |
| Multilingual-E5-base | 278,043,648 | intfloat/multilingual-e5-base |
| BioSimCSE -base | 108,232,704 | kamalkraj/  BioSimCSE-BioLinkBERT-BASE |
| BioClinicalBERT | 108,310,272 | emilyalsentzer/Bio_ClinicalBERT |
| ClinicalBERT | 134,734,080 | medicalai/ClinicalBERT |
| MedEmbed-base | 109,482,240 | abhinand/MedEmbed-base-v0.1 |
| BiomedNLP-BiomedBERT-base | 109,482,240 | microsoft/BiomedNLP-BiomedBERT-base-uncased-abstract-fulltext |
| Bluebert | 109,482,240 | bionlp/bluebert_pubmed_mimic_uncased_L-12_H-768_A-12 |
| PubMedBert-sts | 109,482,240 | bcwarner/PubMedBERT-base-uncased-sts-combined |

## Preprocessing efficiency

**Supplementary Figure 2** illustrates the effectiveness of the preprocessing step, which serves as the initial stage of our framework designed to refine and streamline the raw data. As detailed in the ***Framework*** of the main manuscript and ***Details of preprocessing*** in **Supplementary Materials**, report segments were filtered based on the presence of target-related keywords to ensure clinical relevance. The impact of this process is evident in both the volume and the density of the data: among the 288,076 raw reports analyzed, the filtering process identified that 76% were unrelated to recurrence and 51% were unrelated to metastasis, allowing for the exclusion of a substantial amount of extraneous information. Furthermore, for the reports that remained, the preprocessing stage significantly condensed the textual content. As shown in the histograms of text length distributions in the left subfigure, the average text length was reduced by 72.21 characters for recurrence and 68.16 characters for metastasis. These figures represent a substantial reduction of 53.49% and 42.85% relative to the original report lengths, respectively. By effectively isolating pertinent information and decreasing the overall data burden, this preprocessing stage ensures that subsequent components of the framework can focus on the most relevant clinical features with high computational efficiency.

**Supplementary Figure 2.** Empirical cumulative distribution functions (ECDFs) of report length (character count, log scale) before and after preprocessing. (*Left*) Overall ECDF for raw reports and keyword-filtered reports for recurrence and metastasis targets, showing that preprocessing excluded 76% of raw reports for recurrence and 51% for metastasis. (*Right*) ECDFs stratified by target, comparing raw (dashed) and filtered (solid) report lengths for recurrence (blue) and metastasis (orange), demonstrating a mean length reduction of 53.49% and 42.85%, respectively.

## Description of Revisions 1 and 2

**Supplementary Table 2** shows the data counts for Revisions 1 and 2 used for training and validation. The right side of the table, labeled “Raw,” represents the total count of all reviewed samples. The leftmost column, “Drop Duplicates,” signifies the total count of samples remaining after eliminating those with identical preprocessed text. “Uni.” and “D.C.” stand for unique and double-checked, respectively. Unique refers to the distinct (nonduplicated) text entries within each revision, while double-checked indicates the number of text entries that were reviewed twice with the same text. Following the methodology for dataset construction, we trained our model using the samples shaded with a green background. The evaluation was performed using the samples shaded with a blue background during model cross-validation.

## Consistency of Revision 3's distribution

**Supplementary Table 4** represents the result of the MMD tests with Revision 3 samples depending on each class. Each cell contains two values separated by a forward slash (/), representing the sample size and the computed $p$-value. Across all classes, the $p$-values consistently exceeded the significance level of 0.05. These results statistically confirm that the sampled subsets accurately preserve the distributional characteristics of the entire population without significant bias.

Supplementary Table 4. Results of Linear MMD tests for Revision 3 samples.

| Target | Negative | Uncertain | Positive |
| --- | --- | --- | --- |
| Recurrence | 106 / 0.618 | 92 / 0.235 | 102 / 0.490 |
| Metastasis | 105 / 0.706 | 93 / 0.427 | 102 / 0.531 |

## Unused negative samples

In this section, we randomly sampled from the unused negatives to display examples of reports that were excluded from the learning pipeline. We demonstrated two strategies for eliminating unrelated negatives: filtering during preprocessing and exclusion during presumed negative sampling.

First, **Supplementary Table 5** presents eight representative negative-class report texts of varying lengths that were excluded during preprocessing. Because these texts contain no target keywords related to recurrence or metastasis ("recur" or "metas"), they yielded no output after keyword filtering and were therefore designated as presumed negatives and removed from subsequent classification steps. The examples illustrate that such reports describe postoperative changes, fluid collections, and unrelated incidental findings rather than oncologic events.

Second, **Supplementary Table 6** presents longitudinal preprocessing examples from a representative patient who underwent PH or intraop RFA. The table follows the format of **Table 4** in the main manuscript, displaying the raw report text, the keyword-filtered text, and the extracted recurrence and metastasis segments for each inspection date. Entries with no filtered output confirm that reports lacking target keywords are correctly excluded as presumed negatives, while reports containing relevant terms are retained and passed to the classification pipeline.

**Supplementary Table 7** presents filtered text examples comparing samples drawn from homogeneous negative clusters versus mixed-class clusters identified during the presumed negative labeling stage. The column headers denote the class composition of each cluster. The final major row contains samples exclusively from negative-only clusters, whereas the upper rows contain samples from clusters with mixed class composition (i.e., clusters containing Negative, Positive, and Uncertain cases). Because samples from mixed-class clusters cannot be confidently assumed to be negative without expert verification—as they may contain oncologically relevant findings described with varying linguistic patterns—they were excluded from the weak-negative pool. In contrast, samples drawn from negative-only clusters consist of comparatively straightforward and unambiguous reports that can be safely incorporated as weak negatives during semi-supervised training without requiring additional clinician review.

Supplementary Table 5. Unused negative samples.

Raw texts

1. Air containing fluid collection around the hepaticojejunostomy site with intrahepatic extension.
 --> R/O complicated fluid collection.
2. Mild residual bile duct dilatation.

Increased the size of low attenuation lesion in the abdominal aorta, compared to 2015-11-14, r/o thrombus.
S/P IVC stent insertion, collapsed distal part of the IVC, r/o secondary finding due to general condition.
 - rec) CT low extremity angiography, or enhanced abdomen CT.

1. Decreased amount of bilateral pleural effusion.
 Nearly disappeared passive atelectasis in BLLs.
 Disappeared post-op. subphrenic pneumoperitoneum
---> R/O improvement of post-op. change

1. Decreased attenuation of the right sided liver graft.
2. No evidence of major complication, otherwise.

No evidence of postop. unusual finding around op. site.

1. No change in a 1.5-cm adenoma arising from the left adrenal gland.
2. Perfusion decrease in the right lobe of liver, probably due to newly developed thrombus (1.5 cm) in the aorta (SE 4 IM 12).

1. Newly developed abscesses in left pararenal space.
2. No significant change of left urinary tract obstruction probably due to sigmoid colon diverticulitis.
3. Increased amount of ascites.
4. Right pleural effusion.

1. S/P right anterior sectionectomy.

2. Decreased postop. fluid collection.

3. No new arterial enhancing HCC.

Supplementary Table 6. Preprocessing examples of postoperative CT reports from a representative patient received PH or intraop RFA operation.

| Inspection Date | Raw texts | Filtered texts | Recurrence | Metastasis |
| --- | --- | --- | --- | --- |
| 20151130 | 1. No change, R/O cholangiocarcinoma in the segment VIII. 2. Celiac stenosis. |  |  |  |
| 20151206 | 1. Small amount fluid collection around the liver resection margin and right transverse abdominal wound.  2. Right pleural effusion and atelectasis.  - Postop. change, more likely. |  |  |  |
| 20160305 | 1. Tumorectomy, segment VIII for cholangiocarcinoma. 2. Low density area around op. bed.  --> Postop. change is more likely than recurred tumor. 3. Decreased postop. fluid collection, op. bed. | 1. Tumorectomy, segment VIII for cholangiocarcinoma. 2. Low density area around op. bed. Postop. change is more likely than recurred tumor. 3. Decreased postop. fluid collection, op. bed. | ['Low density area around op. bed.\nPostop. change is more likely than recurred tumor.'] |  |
| 20160411 | 1) No evidence of bone metastasis 2) Degenerative change in T-L spine and right ischium 3) Osteoarthritis in both acromioclavicular, both knee and left ankle joints | 1) No evidence of bone metastasis 2) Degenerative change in T-L spine and right ischium 3) Osteoarthritis in both acromioclavicular, both knee and left ankle joints |  | ['1) No evidence of bone metastasis'] |
| 20160412 | 1) Focal hypermetabolic lesion in the partial hepatectomy site:  R/O Recurred tumor 2) Biopsy proven metastatic adenocarcinoma in the right buccal area 3) Mild hypermetabolic enlarged lymph node in the right cardiophrenic angle:  Reactive lymph node hyperplasia vs. metastatic lymph node  4) Reactive lymph node hyperplasia in both cervical level Ⅱ areas 5) Degenerative change in the interspinous space of L3-4 | 1) Focal hypermetabolic lesion in the partial hepatectomy site: Rule Out Recurred tumor 2) Biopsy proven metastatic adenocarcinoma in the right buccal area 3) Mild hypermetabolic enlarged lymph node in the right cardiophrenic angle: Reactive lymph node hyperplasia vs. metastatic lymph node  4) Reactive lymph node hyperplasia in both cervical level Ⅱ areas 5) Degenerative change in the interspinous space of L3-4 | ['1) Focal hypermetabolic lesion in the partial hepatectomy site:\nRule Out Recurred tumor'] | [' Biopsy proven metastatic adenocarcinoma in the right buccal area', ' Mild hypermetabolic enlarged lymph node in the right cardiophrenic angle:\nReactive lymph node hyperplasia vs. metastatic lymph node '] |
| 20160715 | 1. Neither local recurrence nor distant metastasis. | 1. Neither local recurrence nor distant metastasis. | ['1. Neither local recurrence nor distant metastasis.'] | ['1. Neither local recurrence nor distant metastasis.'] |
| 20160804 | #Compared to 2016-04-12 PET/CT; 1) Increased extent of hypermetabolic lesion in liver;  R/O Recurred cholangiocellular carcinoma 2) Aggravated metastatic tumor in right buccal area 3) No signifiant [sic] change of hypermetabolic lymph node in right cardiophrenic angle;  R/O metastasis 4) New focal hypermetabolic lesion in left lower posterior chest wall:  R/O metastasis | #Compared to 2016-04-12 PET/CT; 1) Increased extent of hypermetabolic lesion in liver; Rule Out Recurred cholangiocellular carcinoma 2) Aggravated metastatic tumor in right buccal area 3) No signifiant [sic] change of hypermetabolic lymph node in right cardiophrenic angle; Rule Out metastasis 4) New focal hypermetabolic lesion in left lower posterior chest wall: Rule Out metastasis | [' Increased extent of hypermetabolic lesion in liver;\nRule Out Recurred cholangiocellular carcinoma'] | [' Aggravated metastatic tumor in right buccal area', ' No signifiant [sic] change of hypermetabolic lymph node in right cardiophrenic angle;\nRule Out metastasis', ' New focal hypermetabolic lesion in left lower posterior chest wall:\nRule Out metastasis'] |
| 20161026 | 1. Small amount of pleural effusion with pleural change in right lower hemithorax, postop. change. 2. No change of small ovoid lymph node in right cardiophrenic angle area.  --> Reactive hyperplasia, more likely. 3. No definite evidence of metastasis in thorax. | 1. Small amount of pleural effusion with pleural change in right lower hemithorax, postop. change. 2. No change of small ovoid lymph node in right cardiophrenic angle area. Reactive hyperplasia, more likely. 3. No definite evidence of metastasis in thorax. |  | ['No definite evidence of metastasis in thorax.'] |
| 20161026 | 1. Soft tisue [sic] density around op. bed.  R/O recurred tumor. 2. No change of bile duct dilatation in the remnant liver. | 1. Soft tisue [sic] density around op. bed. Rule Out recurred tumor. 2. No change of bile duct dilatation in the remnant liver. | ['1. Soft tisue [sic] density around op. bed.\nRule Out recurred tumor.'] |  |

Supplementary Table 7

| Class | Filtered texts |
| --- | --- |
| P. & N. | 1. A 7 cm sized lobulating contoured mass in S3: Primary tumor such as cholangiocarcinoma, most likely. Rule Out metastasis from unknown primary site.  Multiple metastatic lymphadenopathy around the left gastric artery, celiac axis and common hepatic artery, one of which closely abut to the common hepatic artery and portal vein. |
|  | 1. Hypermetabolic lesion at right subphrenic space (SUVmax: 2.8)- APCT commented lesion: Metastasis, most likely. Suspicious hypermetabolic nodular lesion in the pelvic cavity (#208): Metastasis, more likely. |
|  | 1. Large cholangiocarcinoma most likely in the segment VIII - IV with suspicious middle hepatic vein invasion and multiple lymph node metastases. |
|  | Progression of hepatic and perihepatic metastases. |
|  | 1. Increased tumor burden of pancreas cancer with peritoneal metastasis. |
|  | Equivocal change of hepatic metastases, peritoneal carcinomatosis and left periureteral metastasis causing hydronephrosis. |
|  | 1. Slightly increased large pancreatic cancer with invasion of the adjacent structures and peritoneal metastasis compared with 2021-7-18. |
|  | 1. Equivocal change in tumor burden (peritoneal carcinomatosis, colon metastasis along the hepatic flexure, suspicious metastasis involving the duodenum, bilateral periureteric metastases). Slightly more prominent bilateral hydronephrosis, probably due to bilateral periureteric metastases. |
|  | 1. Markedly increased extent of multifocal bone metastases, up to 10.7 cm extraosseous metastatic mass at left pelvic bone. Newly appeared right hydronephrosis, suspected right periureteric metastasis. No significant change of left hydronephrosis with suspected periureteral metastasis. Equivocal change of retroperitoneal metastatic lymphadenopathy and left adrenal metastasis. |
|  | Metastatic lymph node in paraaortic area  Hypermetabolic nodule in remnant left hepatic lobe: Rule Out Metastasis  A mild hypermetabolic nodular lesion in right abdominal wall: Postoperative change > Metastasis |

| Class | Filtered texts |
| --- | --- |
| N. & U. | 1. Successful RF ablation for the metastasis in S7 without any immediate post-procedural complication. A subcentimeter size ill-defined nodule in S8: Metastasis vs. hemangioma. |
|  | 1. New ill-defined low density focal lesion at liver S7 Rule Out non-specific inflammatory lesion > metastasis. |
|  | 1. A low density lesion in segment VII with peripheral enhancement and a newly developed tiny low density lesion in segment VIII (series# 4 image# 17, 16): Rule Out metastasis, Rule Out abscess, Rule Out complicated hepatic cyst. |
|  | 1. Newly developed three low density lesions in left lobe of liver (Se 2 Im 18,21,22), Rule Out metastasis. |
|  | Metastasis vs. inflammatory lesion in right hepatic dome. |
|  | A newly developed small low attenuating lesion in the right hepatic dome. Rule Out biloma more likely than metastasis. |
|  | Slightly increased size of previously mentioned ill-defined low density mass in the segment III, VI/VII and segment V. Liver metastasis rather than inflammatory lesion. |
|  | Several enlarged LNs in the right obturator station. Rule Out metastatic LNs. Subcentimeter LNs in the left obturator station. Rule Out reactive change vs. metastasis. |
|  | Multiple inflammatory lesions rather than metastases in liver. |
|  | Low density lesions in liver segment IV/III subcapsular portion (IM 18): Ischemic lesion or iatrogenic lesion due to surgical manipulation, more likely than new hepatic metastasis. |
| N. only | Distant metastasis (-). |
|  | Distant metastasis (-) |
|  | Lymph node metastasis (-). Distant metastasis (-). |
|  | Distant metastasis (-) |
|  | 1. A 1.1-cm ill-defined low density in the liver S4: focal fat-deposition rather than metastasis. REC) Short-term Follow Up with CT. |
|  | Regional lymph node metastasis: (-). Distant metastasis: (-). |
|  | Distant metastasis (-). |
|  | Regional lymph node metastasis: absent.  Distant metastasis: absent.  Criteria for lymph node metastasis: >1 cm in short-axis diameter or necrosis |
|  | Regional lymph node metastasis: absent Distant metastasis: absent Criteria for lymph node metastasis: >1 cm in short-axis diameter or necrosis |
|  | 1. Asymmetric enhancing mass in prostate, prominent at left prstate [sic]. No evidence of significant lymphadenopathy nor distant metastasis. |

Supplementary Reference

[S1]	A. Alekseev, E. Tutubalina, V. Malykh, and S. Nikolenko, "Improving unsupervised neural aspect extraction for online discussions using out-of-domain classification," *Journal of Intelligent & Fuzzy Systems,* vol. 39, no. 2, pp. 2487-2496, 2020-08-31 2020, doi: 10.3233/jifs-179908.

[S2]	J. Lim, I. Sa, H. S. Ahn, N. Gasteiger, S. J. Lee, and B. Macdonald, "Subsentence Extraction from Text Using Coverage-Based Deep Learning Language Models," *Sensors,* vol. 21, no. 8, p. 2712, 2021-04-12 2021, doi: 10.3390/s21082712.

[S3]	A. Mullick, S. Pal, T. Nayak, S.-C. Lee, S. Bhattacharjee, and P. Goyal, "Using Sentence-level Classification Helps Entity Extraction from Material Science Literature," in *Proceedings of the Thirteenth Language Resources and Evaluation Conference*, Marseille, France, June 2022: European Language Resources Association, in Proceedings of the Thirteenth Language Resources and Evaluation Conference, pp. 4540-4545. [Online]. Available: https://aclanthology.org/2022.lrec-1.483/

[S4]	C. R. S. Banerji *et al.*, "Clinicians must participate in the development of multimodal AI," *eClinicalMedicine,* vol. 84, 2025, doi: 10.1016/j.eclinm.2025.103252.

[S5]	F. Xie *et al.*, "Development and validation of a pre-trained language model for neonatal morbidities: a retrospective, multicentre, prognostic study," *The Lancet Digital Health,* vol. 7, no. 12, 2025, doi: 10.1016/j.landig.2025.100926.

[S6]	H. Zhang, H. Xu, X. Wang, F. Long, and K. Gao, "A Clustering Framework for Unsupervised and Semi-Supervised New Intent Discovery," *IEEE Transactions on Knowledge and Data Engineering,* vol. 36, no. 11, pp. 5468-5481, 2024-11-01 2024, doi: 10.1109/tkde.2023.3340732.

[S7]	B. C. Warner, Z. Xu, S. Haroutounian, T. Kannampallil, and C. Lu, "Utilizing Semantic Textual Similarity for Clinical Survey Data Feature Selection," in *Findings of the Association for Computational Linguistics: ACL 2025*, 2025-01-01 2025: Association for Computational Linguistics, pp. 502-520, doi: 10.18653/v1/2025.findings-acl.27.

[S8]	L. Mcinnes, J. Healy, N. Saul, and L. Großberger, "UMAP: Uniform Manifold Approximation and Projection," *Journal of Open Source Software,* vol. 3, no. 29, p. 861, 2018-09-02 2018, doi: 10.21105/joss.00861.

[S9]	M. Ester, H.-P. Kriegel, J. Sander, and X. Xu, "A density-based algorithm for discovering clusters in large spatial databases with noise," presented at the Proceedings of the Second International Conference on Knowledge Discovery and Data Mining, Portland, Oregon, 1996.

[S10]	K. Taha, P. D. Yoo, C. Yeun, D. Homouz, and A. Taha, "A comprehensive survey of text classification techniques and their research applications: Observational and experimental insights," *Computer Science Review,* vol. 54, p. 100664, 2024/11/01/ 2024, doi: https://doi.org/10.1016/j.cosrev.2024.100664.

[S11]	*MedEmbed: Medical-Focused Embedding Models*. (2024). [Online]. Available: https://github.com/abhinand5/MedEmbed

[S12]	A. Jamin and A. Humeau-Heurtier, "(Multiscale) Cross-Entropy Methods: A Review," *Entropy,* vol. 22, no. 1, p. 45, 2019-12-29 2019, doi: 10.3390/e22010045.

[S13]	T.-Y. Lin, P. Goyal, R. Girshick, K. He, and P. Dollar, "Focal Loss for Dense Object Detection," *IEEE Transactions on Pattern Analysis and Machine Intelligence,* vol. 42, no. 2, pp. 318-327, 2020-02-01 2020, doi: 10.1109/tpami.2018.2858826.

[S14]	A. v. d. Oord, Y. Li, and O. Vinyals, "Representation Learning with Contrastive Predictive Coding," *ArXiv,* vol. abs/1807.03748, 2018.

[S15]	Y. Si, J. Wang, H. Xu, and K. Roberts, "Enhancing clinical concept extraction with contextual embeddings," *Journal of the American Medical Informatics Association,* vol. 26, no. 11, pp. 1297-1304, 2019, doi: 10.1093/jamia/ocz096.

[S16]	S. Rabanser, S. Günnemann, and Z. C. Lipton, "Failing loudly: an empirical study of methods for detecting dataset shift," in *Proceedings of the 33rd International Conference on Neural Information Processing Systems*. Red Hook, NY, USA: Curran Associates Inc., 2019.

[S17]	M. Lin, H. C. Lucas, and G. Shmueli, "Research Commentary---Too Big to Fail: Large Samples and the p-Value Problem," *Info. Sys. Research,* vol. 24, no. 4, pp. 906–917, 2013, doi: 10.1287/isre.2013.0480.

[S18]	Z. Bai and H. Saranadasa, "EFFECT OF HIGH DIMENSION: BY AN EXAMPLE OF A TWO SAMPLE PROBLEM," *Statistica Sinica,* vol. 6, no. 2, pp. 311-329, 1996.

[S19]	A. Gretton, K. M. Borgwardt, M. J. Rasch, B. Schölkopf, and A. Smola, "A kernel two-sample test," *J. Mach. Learn. Res.,* vol. 13, no. null, pp. 723–773, 2012/3 2012.

[S20]	O. Sener and S. Savarese, "Active Learning for Convolutional Neural Networks: A Core-Set Approach," presented at the International Conference on Learning Representations, 2018, 2018. [Online]. Available: https://openreview.net/forum?id=H1aIuk-RW.

[S21]	T. Wolf *et al.*, "Transformers: State-of-the-Art Natural Language Processing," in *Proceedings of the 2020 Conference on Empirical Methods in Natural Language Processing: System Demonstrations*, 2020-01-01 2020: Association for Computational Linguistics, pp. 38-45, doi: 10.18653/v1/2020.emnlp-demos.6.

[S22]	Narine Kokhlikyan *et al.*, "Captum: A unified and generic model interpretability library for PyTorch," *arXiv,* 2020.

[S23]	M. Khodadad, A. S. Kasmaee, M. Astaraki, and H. Mahyar, "Towards Domain Specification of Embedding Models in Medicine," *arXiv,* 2025-07-25  2025, doi: https://doi.org/10.48550/arXiv.2507.19407.

[S24]	Liang Wang, Nan Yang, Xiaolong Huang, Linjun Yang, Rangan Majumder, and F. Wei, "Multilingual E5 Text Embeddings: A Technical Report," *arXiv,* 2024, doi: https://doi.org/10.48550/arXiv.2402.05672.

[S25]	P. Christen, D. J. Hand, and N. Kirielle, "A Review of the F-Measure: Its History, Properties, Criticism, and Alternatives," *ACM Computing Surveys,* vol. 56, no. 3, pp. 1-24, 2024-03-31 2024, doi: 10.1145/3606367.
